# Supplementary material for: Chiral Indolizinium Salts Derived from 2-Pyridinecarbaldehyde—First Diastereoselective Syntheses of (−)-1-epi-lentiginosine
Source: Molecules. 2023 Apr 25;28(9):3719. doi: 10.3390/molecules28093719 (PMC10180032; doi:10.3390/molecules28093719)

Supporting information

Chiral indolizinium salts derived from 2-pyridinecarbaldehyde. First diastereoselective syntheses of (–)-1-*epi*-lentiginosine

Hisami Rodriguez-Matsui [1], David M. Aparicio [1], María L. Orea [1], Jorge R. Juárez [1], Victor Gómez-Calvario [1], Dino Gnecco [1], Alan Carrasco-Carballo [2], and Joel L. Terán [1,*]

[1]Centro de Química, Instituto de Ciencias, Benemérita Universidad Autónoma de Puebla, Edif. IC-9 Complejo de Ciencias C.U. 72570 Puebla, México

[2]Laboratorio de Elucidación y Síntesis en Química Orgánica, Benemérita Universidad Autónoma de Puebla, C.U., 72570 Puebla, México

Table of Contents

1. General Information…………………………………… ….. ....S2

2. Characterization of Products.… ……….S3

3. ^{1}H and ^{13}C NMR Spectra…….………...S7

4. X-ray crystallography data S28

General Information

All reagents and solvents were purchased from commercial sources. The ^{1}H and ^{13}C spectra were determined with a Bruker Avance III Spectrometer ($CDCl_3$ or CD_3OD solvents) operating at 500 and 125 MHz, respectively. The chemical shifts were reported in parts per million (ppm), downfield from SiMe4 (δ 0.0) and relative to the signal of chloroform-d (δ 7.26, singlet). Multiplicities were afforded as: s (singlet); d (doublet); t (triplet); q (quartet); dd (doublets of doublet); ddd (doublet of doublets of doublets); or m (multiplets). The number of protons for a given resonance is indicated by nH. Coupling constants were reported as a *J* value in Hz. Thin layer chromatography (TLC) was used to monitor the reaction on Merck 60 F254 precoated silica gel plate (0.2 mm thickness). Optical rotations were determined at room temperature with a Perkin-Elmer 341 polarimeter, using a 1 dm cell with a total volume of 1mL, and are referenced to the D-line of sodium. Mass spectra were recorded with a JEOL Station JMS-700 instrument at a voltage of 70 eV. X-Ray Diffraction analysis was performed on a diffractometer STOE Stadivari using Ag-Kα radiation (λ = 0.56083 Å, AXO micro-source) and equipped with a Dectris Pilatus-100 K detector. Intensities were collected at 295 K, and structures were refined using the current release of SHELXL (2018/3). The products were purified by column chromatography on silica gel 60 (63-200nm).

((2*R*,4*R*)-4-phenyl-2-propyloxazolidin-3-yl)((2*R*,3*S*)-3-(pyridin-2-yl)oxiran-2-yl)methanone, 2a

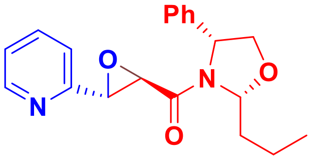

2a

major diastereoisomer

Yellow oil, 85% yield. Major diastereoisomer: ^{1}H NMR (500 MHz, $CDCl_3$) δ 8.33 (d, *J* = 4.8 Hz, 1H), 7.45 (m, 1H), 7.13 (m, 2H), 7.07 (dd, *J* = 7.5, 4.9 Hz, 1H), 7.02 (t, *J* = 7.6 Hz, 2H), 6.90 (m, 2H), 5.51 (dd, *J* = 9.2, 2.5 Hz, 1H), 5.10 (t, *J* = 6.6 Hz, 1H), 4.33 (dd, *J* = 9.1, 6.9 Hz, 1H), 3.89 (dd, *J* = 9.2, 6.3 Hz, 1H), 3.81 (d, *J* = 1.8 Hz, 1H), 3.59 (d, *J* = 1.9 Hz, 1H), 2.15 (dddd, *J* = 16.3, 9.3, 6.2, 2.5 Hz, 1H), 1.69 (dtd, *J* = 14.2, 9.4, 5.3 Hz, 1H), 1.51 (m, 3H), 0.96 (t, *J* = 7.4 Hz, 3H). ^{13}C NMR (125 MHz, $CDCl_3$) δ 165.2, 149.4, 139.2, 136.2, 128.9, 128.8, 127.8, 126.0, 123.2, 121.0, 91.7, 73.7, 60.4, 57.9, 56.4, 35.2, 18.4, 13.7.. **HRMS(ESI):** Calcd for $C_{20}H_{22}N_2O_3$: 338.1630 Found: 338.1629.

(2*S*,3*S*)-2-bromo-3-hydroxy-1-((2*R*,4*R*)-4-phenyl-2-propyloxazolidin-3-yl)-3-(pyridin-2-yl)propan-1-one, 3

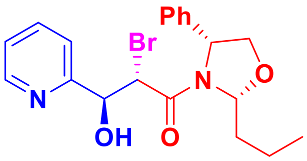

3

White solid, mp = 129 °C, 98% yield. Major rotamer: ^{1}H NMR (500 MHz, $CDCl_3$) δ 8.55 (dt, *J* = 4.0, 0.8 Hz, 1H), 7.72 (m, 1H), 7.72-7.33 (m, 5H), 7.25 (m, 1H), 6.74 (m, 1H), 5.28 (dd, *J* = 9.0, 2.5 Hz, 1H), 5.08 (dd, *J* = 6.6, 4.6 Hz, 1H), 4.99 (br, 1H), 4.73 (d, *J* = 5.5 Hz, 1H), 4.14 (dd, *J* = 8.9, 6.7 Hz, 1H), 3.98 (dd, *J* = 8.9, 4.6 Hz, 1H), 2.17 (m, 1H), 1.70 (m, 2H), 1.49 (m, 2H), 0.99 (t, *J* = 7.5 Hz, 3H). ^{13}C NMR (125 MHz, $CDCl_3$) δ 169.8, 159.4, 138.3, 137.1, 129.1, 126.6, 125.1, 123.3, 122.0, 91.2, 75.9, 73.8, 61.2, 45.6, 34.9, 18.6, 13.9. HRMS(ESI): Calcd for $C_{20}H_{23}BrN_2O_3$: 418.0892 Found: 418.0890.

((2*S*,3*S*)-3-(pyridin-2-yl)oxiran-2-yl)methanol.

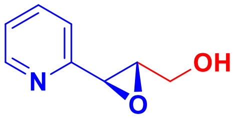

4

Colorless oil, 73% yield. ^{1}H NMR (500 MHz, $CDCl_3$) δ 8.59-8.52 (m, 1H), 7.70 (td, *J* = 7.8, 1.8 Hz, 1H), 7.30 – 7.27 (m, 1H), 7.25 (ddd, *J* = 7.5, 4.9, 1.2 Hz, 1H), 4.08 (d, *J* = 2.1 Hz, 1H), 4.05 (dt, *J* = 12.8, 2.4 Hz, 1H), 3.85 (dd, *J* = 12.8, 4.0 Hz, 1H), 3.39 (dq, *J* = 4.2, 2.0 Hz, 1H). ^{13}C NMR (125 MHz, $CDCl_3$) δ 156.4, 149.4, 137.0, 123.3, 120.3, 61.8, 61.3, 56.0. HRMS(ESI): Calcd for $C_8H_9NO_2$: 151.0633 Found: 151.0630.

((2*S*,3*S*)-3-(pyridin-2-yl)oxiran-2-yl)methyl 4-methylbenzenesulfonate, 5.

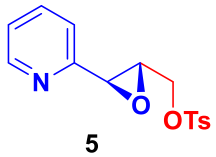

5

Yellow oil, 94% yield. ^{1}H NMR (500 MHz, $CDCl_3$) δ 8.47 (ddd, *J* = 4.9, 1.8, 0.9 Hz, 1H), 7.77 – 7.70 (m, 2H), 7.62 (td, *J* = 7.7, 1.8 Hz, 1H), 7.32 – 7.24 (m, 2H), 7.18 (ddd, *J* = 7.6, 4.9, 1.2 Hz, 1H), 7.15 (dt, *J* = 7.9, 1.1 Hz, 1H), 4.33 (dd, *J* = 11.6, 3.2 Hz, 1H), 4.12 – 3.97 (m, 1H), 3.84 (d, *J* = 2.0 Hz, 1H), 3.38 (ddd, *J* = 5.9, 3.1, 1.9 Hz, 1H), 2.36 (s, 3H). ^{13}C NMR (125 MHz, $CDCl_3$) δ 153.9, 148.3, 144.2, 136.1, 131.5, 129.0, 127.0, 122.6, 119.5, 68.2, 56.7, 55.3, 20.7. HRMS(ESI): Calcd for $C_{15}H_{15}NO_4S$: 305.0722 Found: 305.0721.

Major diastereoisomer (1*R*,2*S*)-1-chloro-2-hydroxy-2,3-dihydro-1H-indolizin-4-ium 4-methylbenzenesulfonate, 6a.

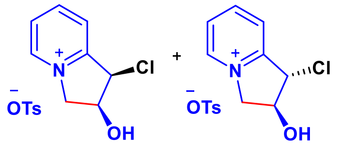

6a **6b**
(*dr*= 81:19)

Black oil, quantitative yield. Inseparable diastereomeric mixture. Major diastereoisomer: ^{1}H NMR (500 MHz, $CDCl_3$) δ 9.13 (d, *J* = 6.0 Hz, 1H), 8.66 (td, *J* = 7.9, 1.3 Hz, 1H), 8.25 (d, *J* = 8.0 Hz, 1H), 8.14 (t, *J* = 6.9 Hz, 1H), 7.48 (d, *J* = 8.0 Hz, 2H), 7.12 (d, *J* = 7.9 Hz, 2H), 5.94 (d, *J* = 4.8 Hz, 1H), 4.97 (dd, *J* = 13.3, 4.6 Hz, 1H), 4.89 (td, *J* = 4.6, 2.7 Hz, 1H), 4.79 (dd, *J* = 13.4, 2.9 Hz, 1H), 2.29 (s, 3H). ^{13}C NMR (125 MHz, $CDCl_3$) δ 154.8, 147.2, 146.0, 143.0, 138.2, 128.6, 128.0, 125.9, 125.4, 69.8, 63.7, 61.2, 21.3. HRMS(ESI): Calcd for $C_{15}H_{16}ClNO_4S$: 341.0488 Found: 341.0486.

Major diastereoisomer (1*R*,2*S*)-1,2-dihydroxy-2,3-dihydro-1*H*-indolizin-4-ium 4-methylbenzenesulfonate, 10a.

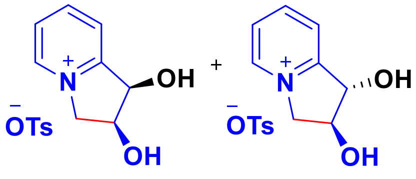

10a **10b**
(*dr*= 81:19)

Black oil, quantitative yield. Inseparable diastereomeric mixture. Major diastereoisomer: ^{1}H NMR (500 MHz, $CDCl_3$) δ 8.70 (d, *J* = 5.9 Hz, 1H), 8.46 (t, *J* = 7.7 Hz, 1H), 8.03 (d, *J* = 8.0 Hz, 1H), 7.88 (t, *J* = 6.7 Hz, 1H), 7.58 (d, *J* = 7.2 Hz, 2H), 7.25 (d, *J* = 6.7 Hz, 2H), 5.65 – 5.36 (m, 1H), 4.81 (m, 3H), 2.29 (s, 3H). ^{13}C NMR (125 MHz, $CDCl_3$) δ 156.7, 146.3, 142.4, 141.4, 139.5, 129.4, 127.2, 125.3, 124.6, 74.2, 70.4, 63.2, 20.5. HRMS(ESI): Calcd for $C_{15}H_{17}ClNO_5S$: 323.0827 Found: 323.0825.

(1*R*,2*S*,8*aS*)-1-chloro-1,2,3,5,8,8a-hexahydroindolizin-2-ol, 11a.

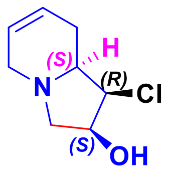

11a

White solid, mp = 140°C, 74% yield. ^{1}H NMR (500 MHz, $CDCl_3$) δ 5.78 (ddd, *J* = 10.0, 5.3, 2.4 Hz, 1H), 5.67 (dtd, *J* = 10.1, 2.9, 1.4 Hz, 1H), 4.51 (dt, *J* = 16.9, 5.2 Hz, 2H), 3.48 (dq, *J* = 16.3, 2.3 Hz, 1H), 3.14 (dd, *J* = 10.6, 4.3 Hz, 1H), 2.89 (br, 1H), 2.81 – 2.66 (m, 2H), 2.56 (ddq, *J* = 14.9, 7.8, 2.4 Hz, 1H), 2.46 (br, 1H), 2.10 (dd, *J* = 17.0, 4.4 Hz, 1H). ^{13}C NMR (125 MHz, $CDCl_3$) δ 124.7, 124.5, 70.4, 66.7, 61.3, 60.1, 52.4, 28.0. HRMS(ESI): Calcd for $C_8H_{12}ClNO$: 173.0607 Found: 173.0605.

(1*S*,2*S*,8*aR*)-1-chloro-1,2,3,5,8,8a-hexahydroindolizin-2-ol, 11b.

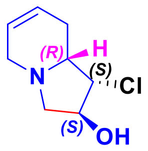

11b

White solid, mp = 136 °C, 14% yield. ^{1}H NMR (500 MHz, $CDCl_3$) δ 5.80 (ddd, *J* = 9.9, 5.3, 2.4 Hz, 1H), 5.69 (dddd, *J* = 9.9, 4.4, 2.9, 1.5 Hz, 1H), 4.54 (ddd, *J* = 6.5, 4.6, 1.5 Hz, 1H), 4.16 (dd, *J* = 4.8, 1.5 Hz, 1H), 3.74 (dd, *J* = 10.5, 6.7 Hz, 1H), 3.52 (ddt, *J* = 16.1, 4.4, 2.1 Hz, 1H), 2.93 (ddd, *J* = 15.9, 4.7, 2.1 Hz, 1H), 2.85 (dt, *J* = 9.4, 4.5 Hz, 1H), 2.53 (dddd, *J* = 17.0, 10.1, 4.5, 2.2 Hz, 1H), 2.25 (d, *J* = 4.6 Hz, 1H), 2.16 – 1.96 (m, 1H).^{13}C NMR (125 MHz, $CDCl_3$) δ 124.4, 78.6, 67.6, 61.4, 60.5, 52.6, 27.6. HRMS(ESI): Calcd for $C_8H_{12}ClNO$: 173.0607 Found: 173.0606.

(1*R*,2*S*,8*aS*)-1-(benzyloxy)-1,2,3,5,8,8a-hexahydroindolizin-2-ol, 13a.

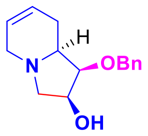

13a

Yellow oil, 80% yield. ^{1}H NMR (500 MHz, $CDCl_3$) δ 7.45 – 7.18 (m, 5H), 5.88 – 5.72 (m, 1H), 5.69 – 5.50 (m, 1H), 4.66 (d, *J* = 11.8 Hz, 1H), 4.59 (d, *J* = 11.8 Hz, 1H), 4.13 (dd, *J* = 9.3, 5.6 Hz, 2H), 3.45 (d, *J* = 16.0 Hz, 1H), 3.24 (d, *J* = 10.7 Hz, 1H), 2.93 (br, 1H), 2.71 (d, *J* = 16.0 Hz, 1H), 2.63 – 2.56 (m, 1H), 2.39 (dd, *J* = 10.7, 7.3 Hz, 1H), 2.18 (dd, *J* = 10.0, 4.1 Hz, 1H), 2.12 – 1.94 (m, 1H). ^{13}C NMR (125 MHz, $CDCl_3$) δ 137.5, 128.5, 127.9, 127.9, 125.4, 124.1, 76.4, 72.5, 70.8, 63.1, 59.2, 52.5, 25.1. HRMS(ESI): Calcd for $C_{15}H_{19}NO_2$: 245.1416 Found: 245.1414.

(1*R*,2*S*,8*aS*)-1-chlorooctahydroindolizin-2-ol, 12a.

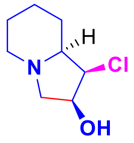

12a

White solid, mp = 148 °C, quantitative yield. ^{1}H NMR (500 MHz, $CDCl_3$) δ 4.42 (dt, *J* = 9.3, 5.2 Hz, 1H), 4.30 (dd, *J* = 5.7, 3.3 Hz, 1H), 2.79 (dt, *J* = 11.3, 3.4 Hz, 1H), 2.65 (dd, *J* = 11.0, 4.7 Hz, 1H), 2.53 (dd, *J* = 11.0, 9.2 Hz, 1H), 2.35 (dt, *J* = 10.8, 3.0 Hz, 1H), 1.98 (td, *J* = 11.9, 3.0 Hz, 1H), 1.63 (dt, *J* = 13.3, 3.4 Hz, 1H), 1.60 – 1.52 (m, 1H), 1.48 – 1.42 (m, 1H), 1.42 – 1.34 (m, 1H), 1.29 (qt, *J* = 13.1, 4.2 Hz, 1H), 1.12 (qt, *J* = 13.1, 4.0 Hz, 1H).^{13}C NMR (125 MHz, $CDCl_3$) δ 69.2, 66.0, 65.7, 57.9, 52.4, 26.1, 24.0, 22.7. HRMS(ESI): Calcd for $C_8H_{14}ClNO$: 175.0764 Found: 175.0763.

(−)-1-*epi* lentiginosine

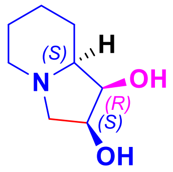

Colorless oil, 87% yield. $[\alpha]_D^{20} = -15.5$ (c 0.3, MeOH). ^{1}H NMR (500 MHz, $CDCl_3$) δ 4.22 (ddd, *J* = 7.4, 5.9, 1.8 Hz, 1H), 3.98 (dd, *J* = 6.0, 4.0 Hz, 1H), 3.69 – 3.61 (m, 2H), 3.04 (dd, *J* = 11.1, 3.5 Hz, 1H), 2.94 (dd, *J* = 10.7, 1.7 Hz, 1H), 2.33 (dd, J = 10.7, 7.0 Hz, 1H), 1.97 – 1.88 (m, 1H), 1.88 – 1.81 (m, 2H), 1.78 – 1.68 (m, 1H), 1.67 – 1.49 (m, 3H). ^{13}C NMR (125 MHz, $CDCl_3$) δ 76.6, 69.7, 62.5, 53.1, 29.7, 25.1, 24.9, 23.8. HRMS(ESI): Calcd for $C_8H_{15}NO_2$: 157.1103 Found: 157.1102.

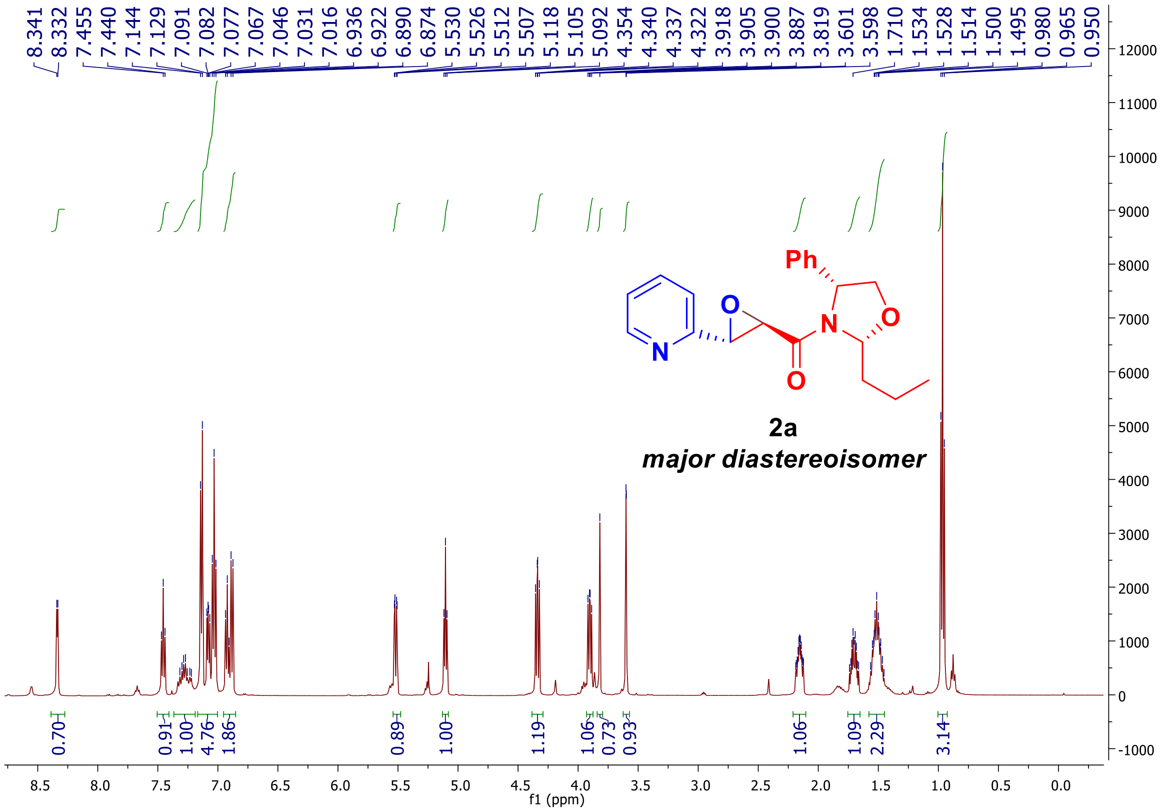
8.341
8.332
7.455
7.440
7.144
7.129
7.091
7.082
7.077
7.067
7.046
7.031
7.016
6.936
6.922
6.890
6.874
5.530
5.526
5.512
5.507
5.118
5.105
5.092
4.354
4.340
4.337
4.322
3.918
3.905
3.900
3.887
3.819
3.601
3.598
1.710
1.534
1.528
1.514
1.500
1.495
0.980
0.965
0.950
Ph
O
N
O
N
O
2a
major diastereoisomer
0.70
0.91
1.00
4.76
1.86
0.89
1.00
1.19
1.06
0.73
0.93
1.06
1.09
2.29
3.14
f1 (ppm)

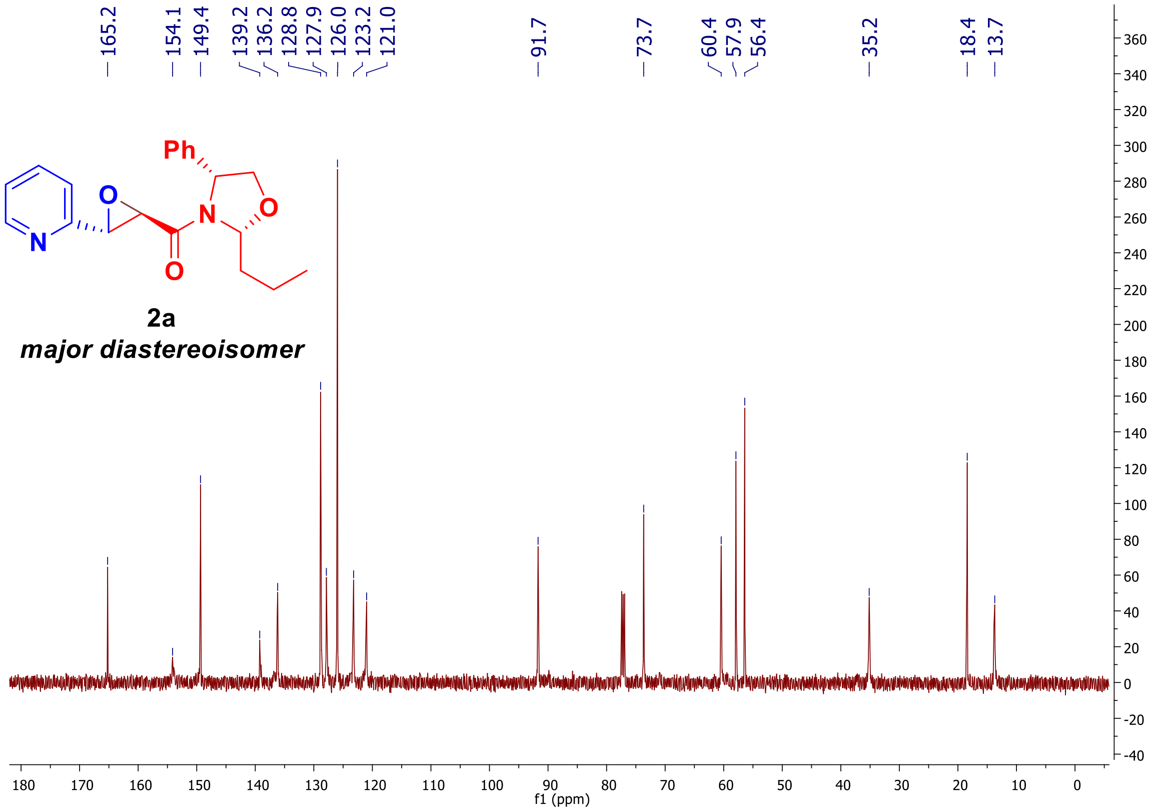
165.2
154.1
149.4
139.2
136.2
128.8
127.9
126.0
123.2
121.0
91.7
73.7
60.4
57.9
56.4
35.2
18.4
13.7
Ph
O
N
O
N
O
2a
major diastereoisomer
f1 (ppm)

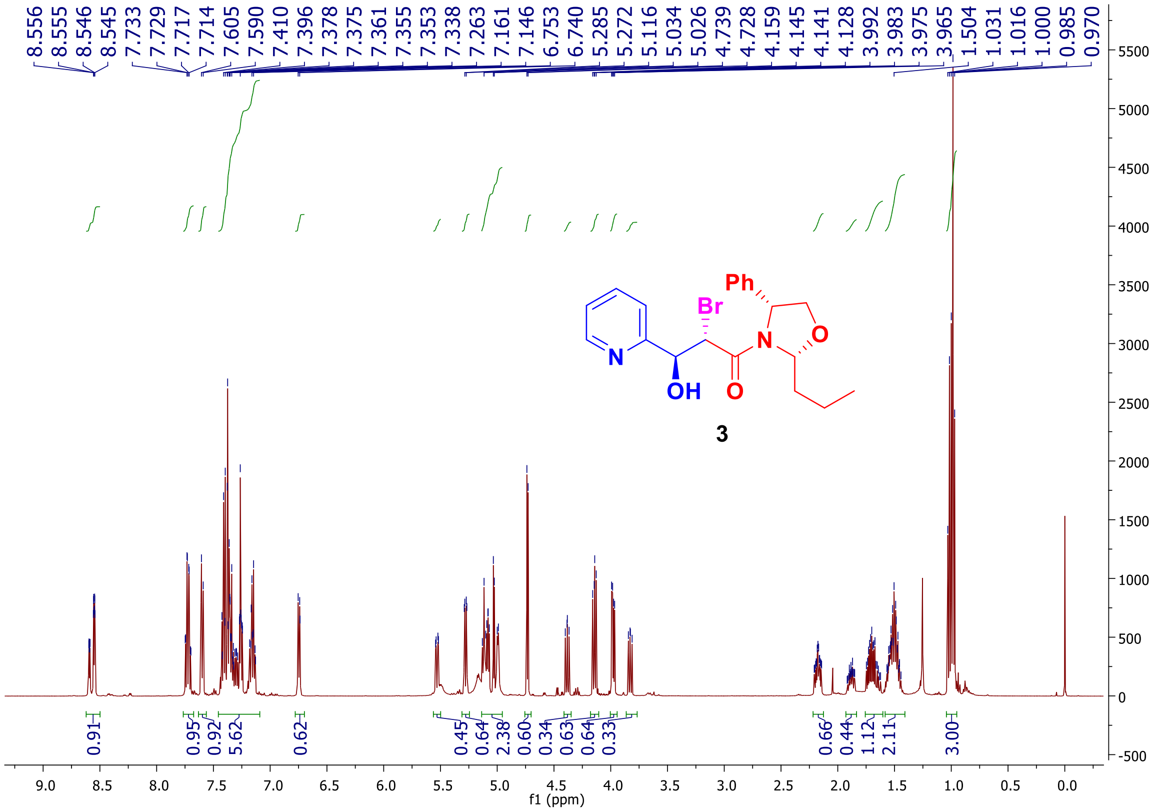
8.556 8.555 8.546 8.545 7.733 7.729 7.717 7.714 7.605 7.590 7.410 7.396 7.378 7.375 7.361 7.355 7.353 7.338 7.263 7.161 7.146 6.753 6.740 5.285 5.272 5.116 5.034 5.026 4.739 4.728 4.159 4.145 4.141 4.128 3.992 3.983 3.975 3.965 1.504 1.031 1.016 1.000 0.985 0.970
0.91 0.95 0.92 5.62 0.62 0.45 0.64 2.38 0.60 0.34 0.63 0.64 0.33 0.66 0.44 1.12 2.11 3.00
Ph
Br
N
OH
O
N
O
3
f1 (ppm)

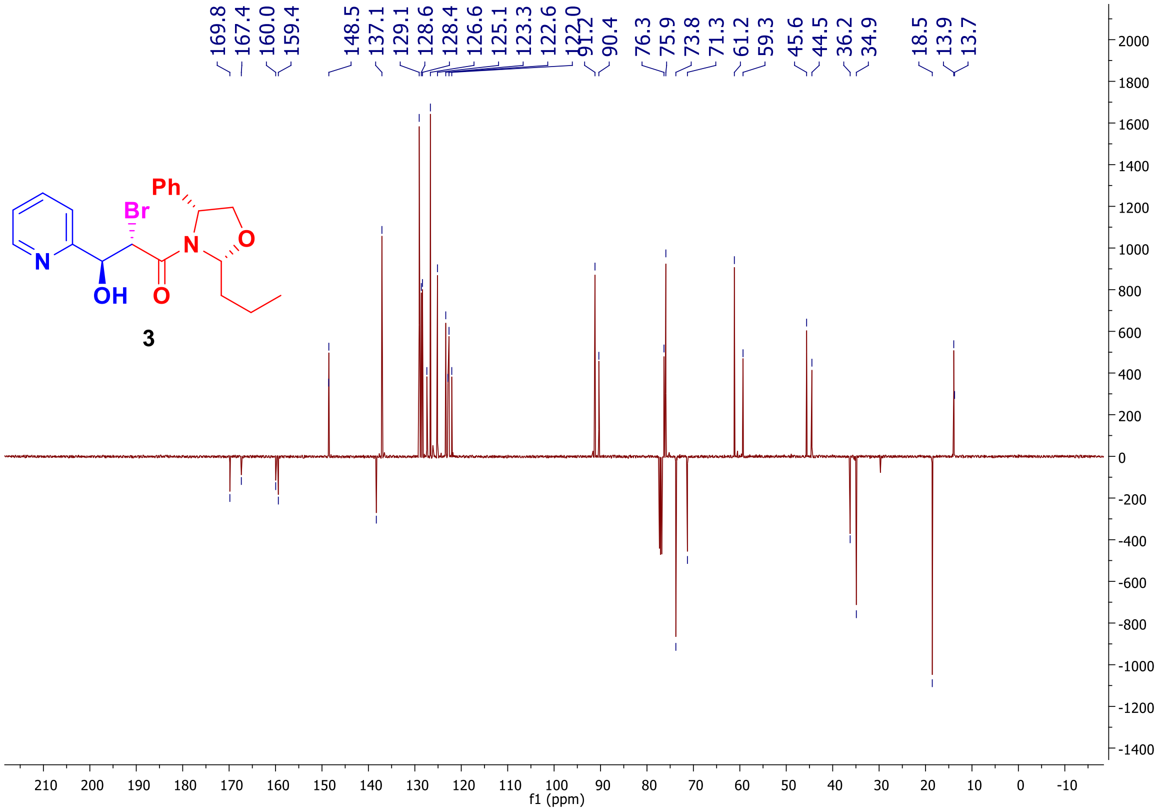
169.8 167.4 160.0 159.4 148.5 137.1 129.1 128.6 128.4 126.6 125.1 123.3 122.6 122.0 91.2 90.4 76.3 75.9 73.8 71.3 61.2 59.3 45.6 44.5 36.2 34.9 18.5 13.9 13.7
Ph
Br
N
OH
O
N
O
3
f1 (ppm)

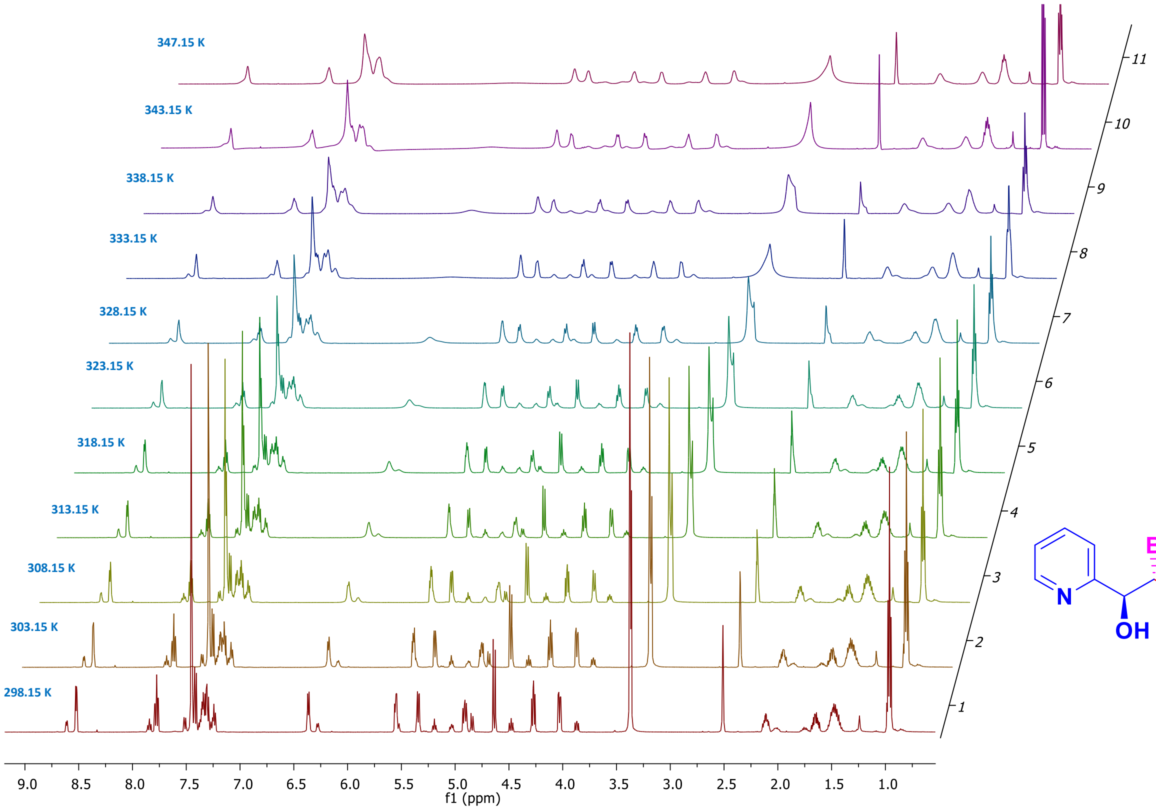

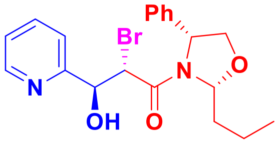

3

Variable temperature NMR experiments.

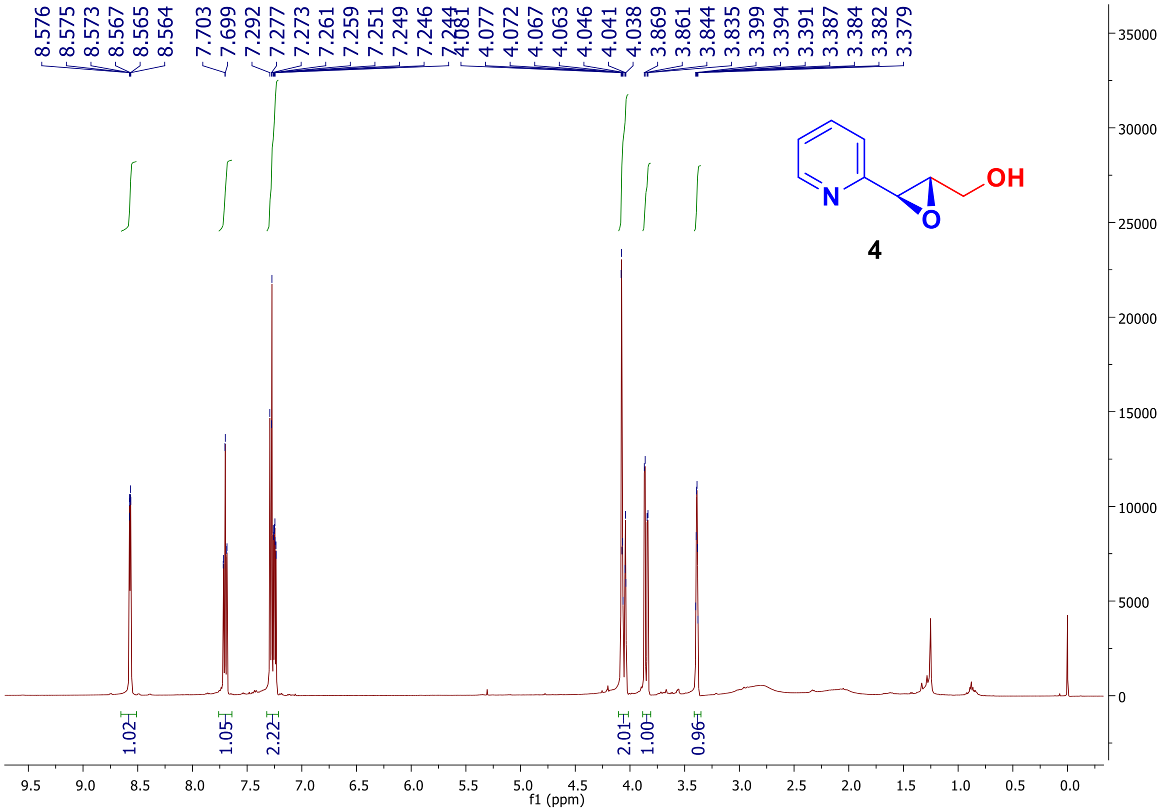
8.576
8.575
8.573
8.567
8.565
8.564
7.703
7.699
7.292
7.277
7.273
7.261
7.259
7.251
7.249
7.246
7.244
4.081
4.077
4.072
4.067
4.063
4.046
4.041
4.038
3.869
3.861
3.844
3.835
3.399
3.394
3.391
3.387
3.384
3.382
3.379
N
O
OH
4
1.02
1.05
2.22
2.01
1.00
0.96
f1 (ppm)

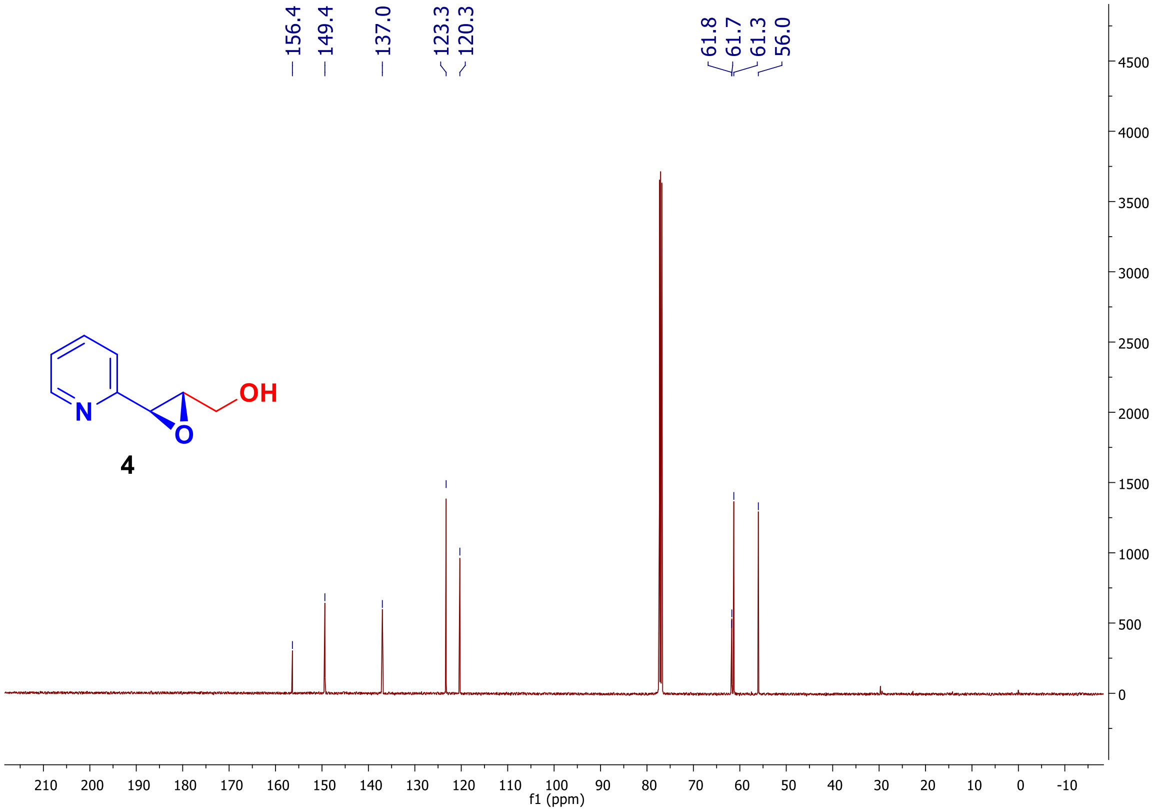
156.4
149.4
137.0
123.3
120.3
61.8
61.7
61.3
56.0
N
O
OH
4
f1 (ppm)

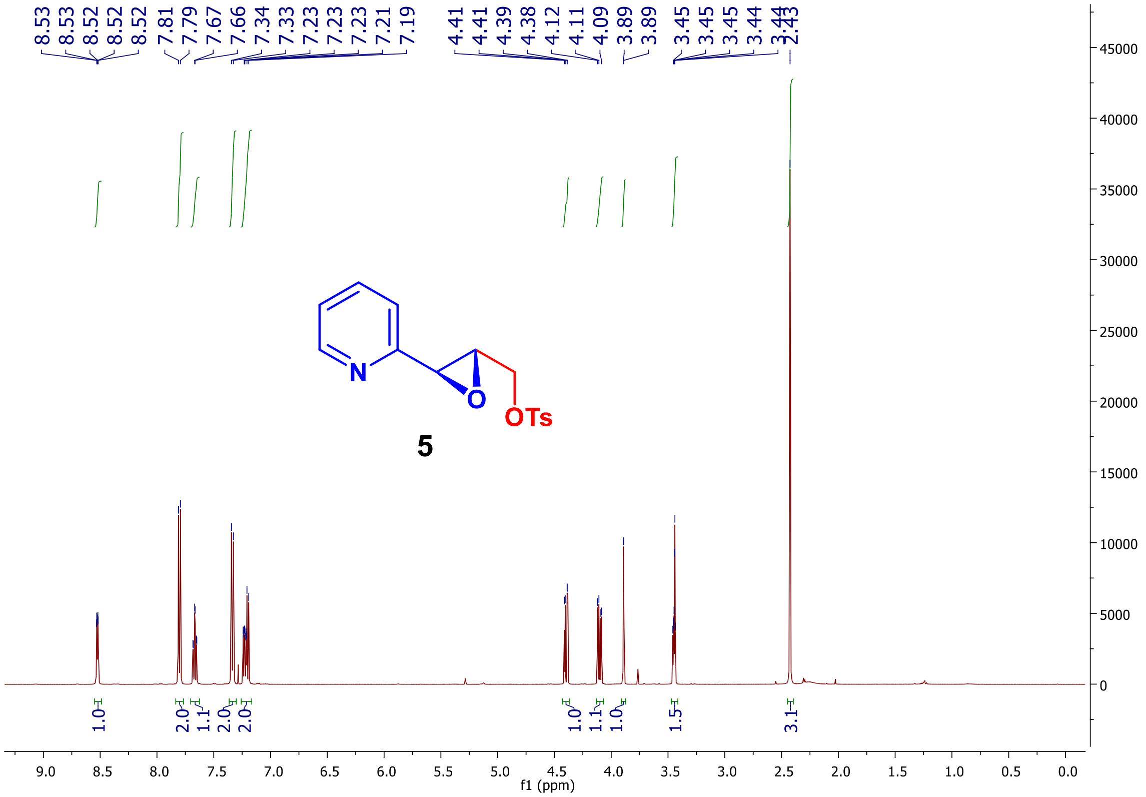

8.53 8.53 8.52 8.52 8.52 7.81 7.79 7.67 7.66 7.34 7.33 7.23 7.23 7.23 7.21 7.19 4.41 4.41 4.39 4.38 4.12 4.11 4.09 3.89 3.89 3.45 3.45 3.45 3.44 3.44 2.43
N
O
OTs
5
1.0 2.0 1.1 2.0 2.0 1.0 1.1 1.0 1.5 3.1
f1 (ppm)

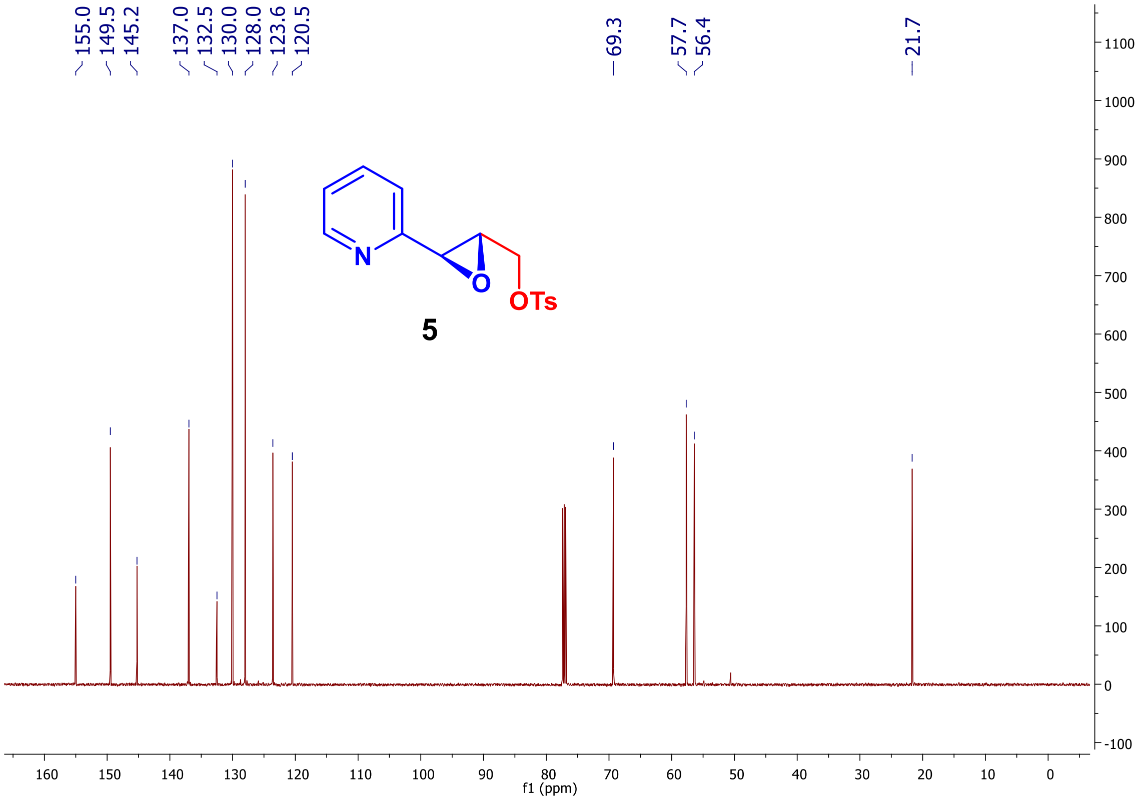

155.0 149.5 145.2 137.0 132.5 130.0 128.0 123.6 120.5 69.3 57.7 56.4 21.7
N
O
OTs
5
f1 (ppm)

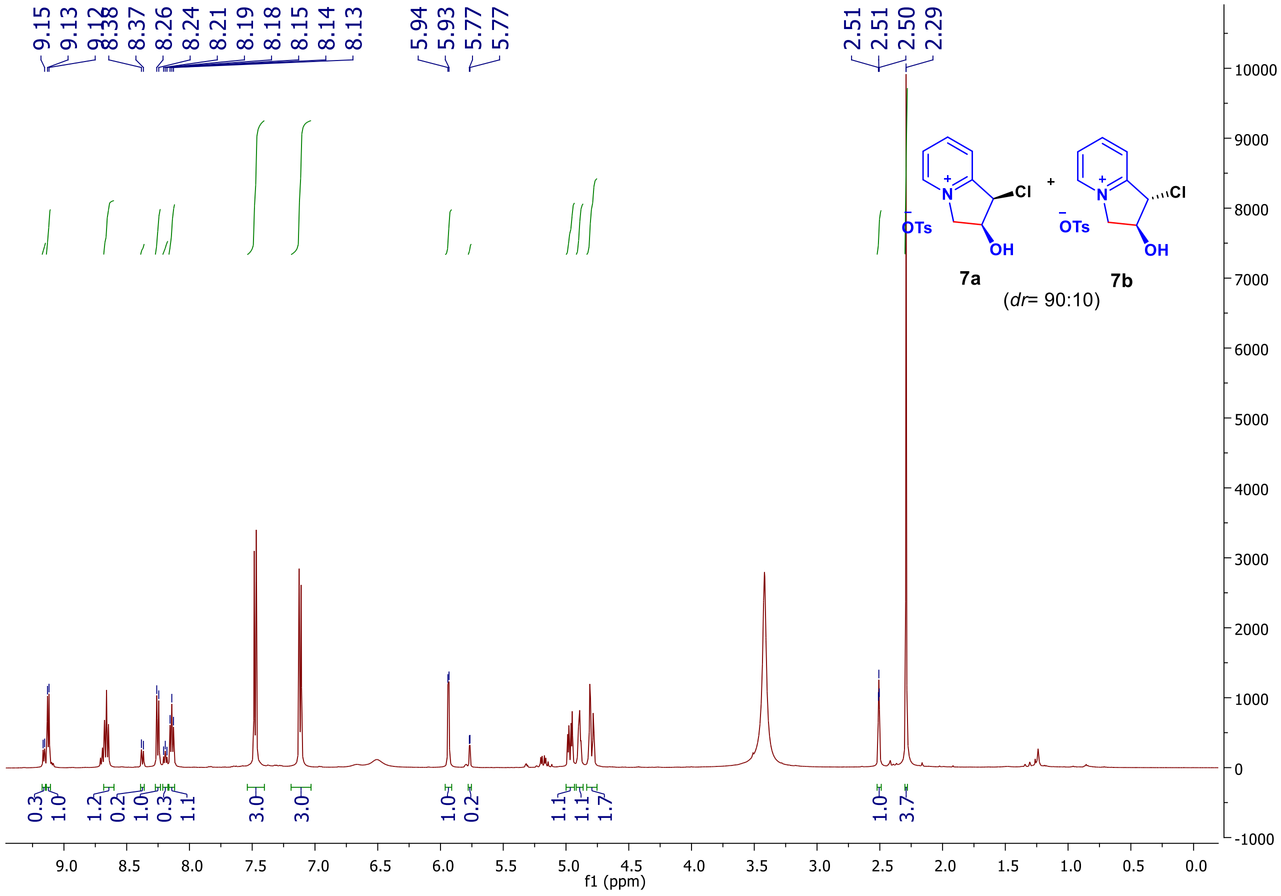

9.15
9.13
9.12
8.38
8.37
8.26
8.24
8.21
8.19
8.18
8.15
8.14
8.13
5.94
5.93
5.77
5.77
2.51
2.51
2.50
2.29
7a
7b
(dr= 90:10)
OTs
Cl
OH
0.3
1.0
1.2
0.2
1.0
0.3
1.1
3.0
3.0
1.0
0.2
1.1
1.1
1.7
1.0
3.7
f1 (ppm)

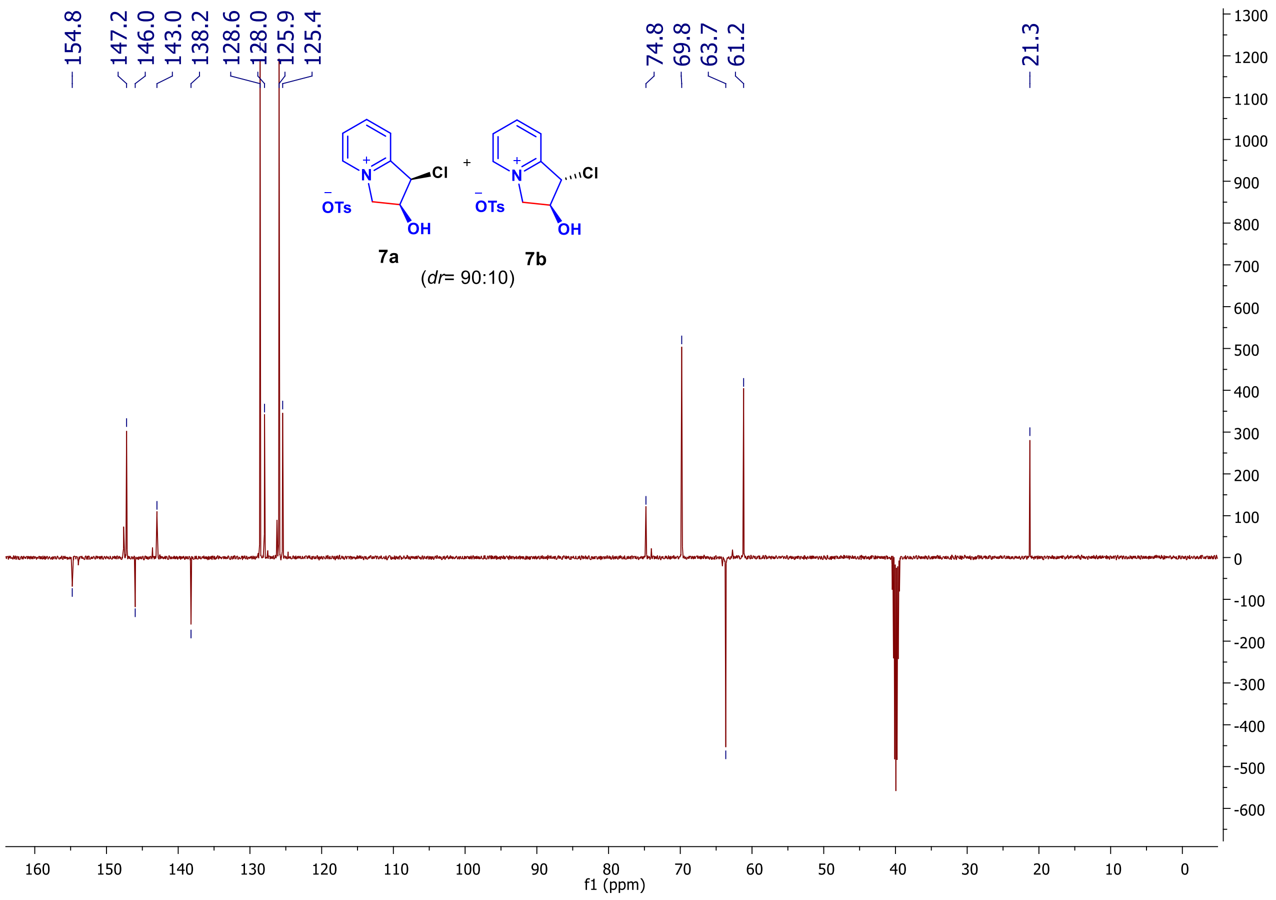

154.8
147.2
146.0
143.0
138.2
128.6
128.0
125.9
125.4
74.8
69.8
63.7
61.2
21.3
7a
7b
(dr= 90:10)
f1 (ppm)

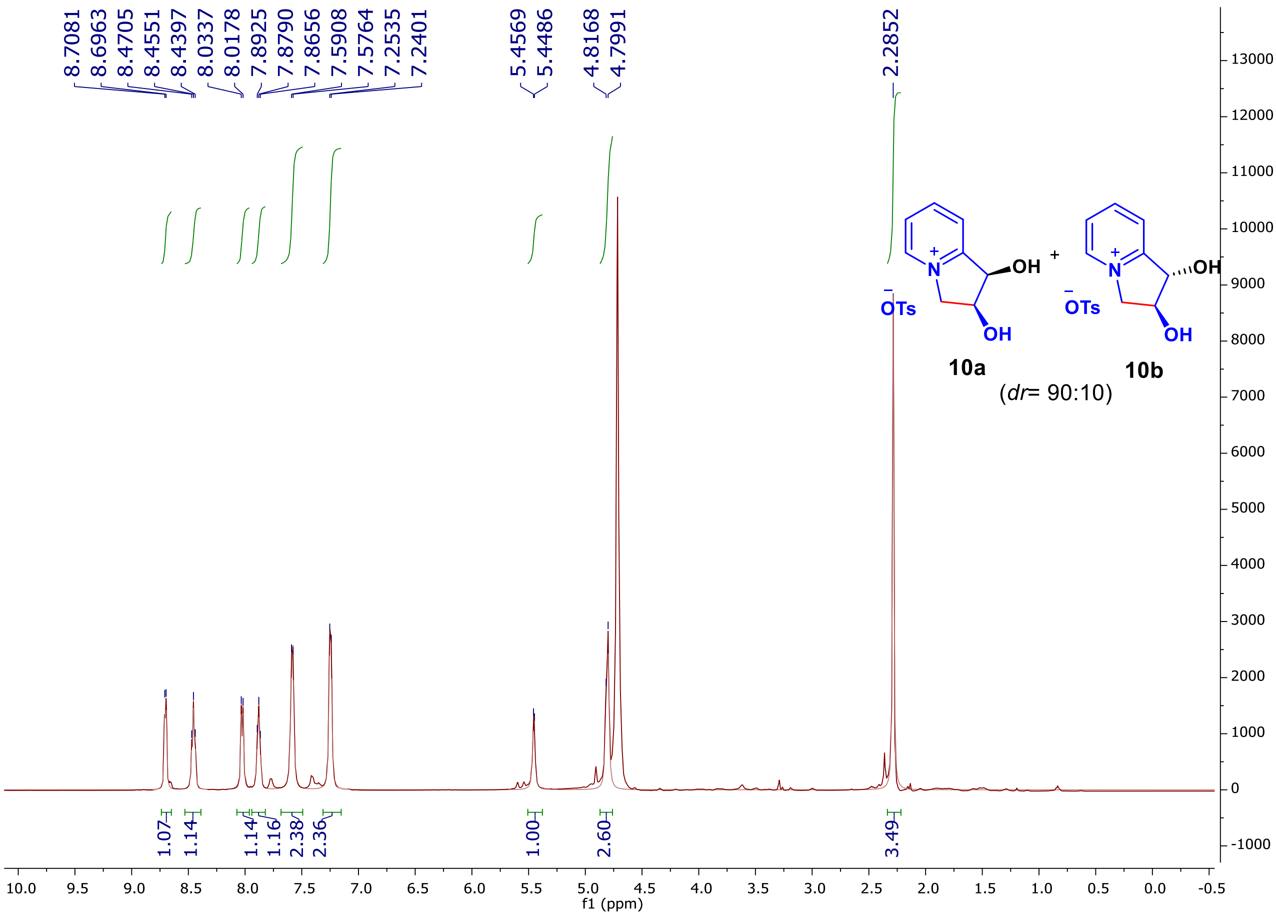
8.7081
8.6963
8.4705
8.4551
8.4397
8.0337
8.0178
7.8925
7.8790
7.8656
7.5908
7.5764
7.2535
7.2401
5.4569
5.4486
4.8168
4.7991
2.2852
1.07
1.14
1.14
1.16
2.38
2.36
1.00
2.60
3.49
f1 (ppm)
10a
10b
(dr= 90:10)
OTs
OH

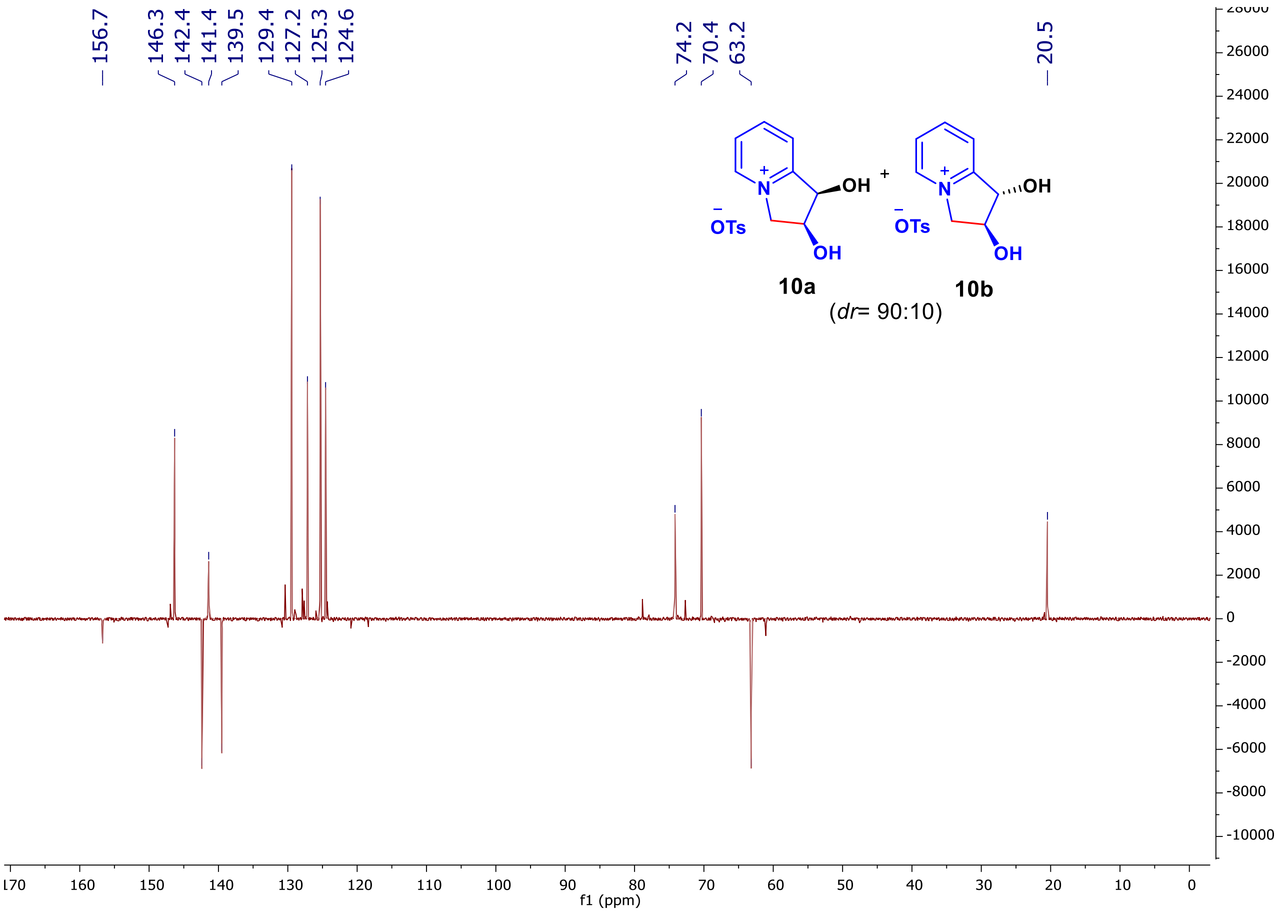

156.7
146.3
142.4
141.4
139.5
129.4
127.2
125.3
124.6
74.2
70.4
63.2
20.5
OTs
OH
OH
10a
10b
(dr= 90:10)
f1 (ppm)

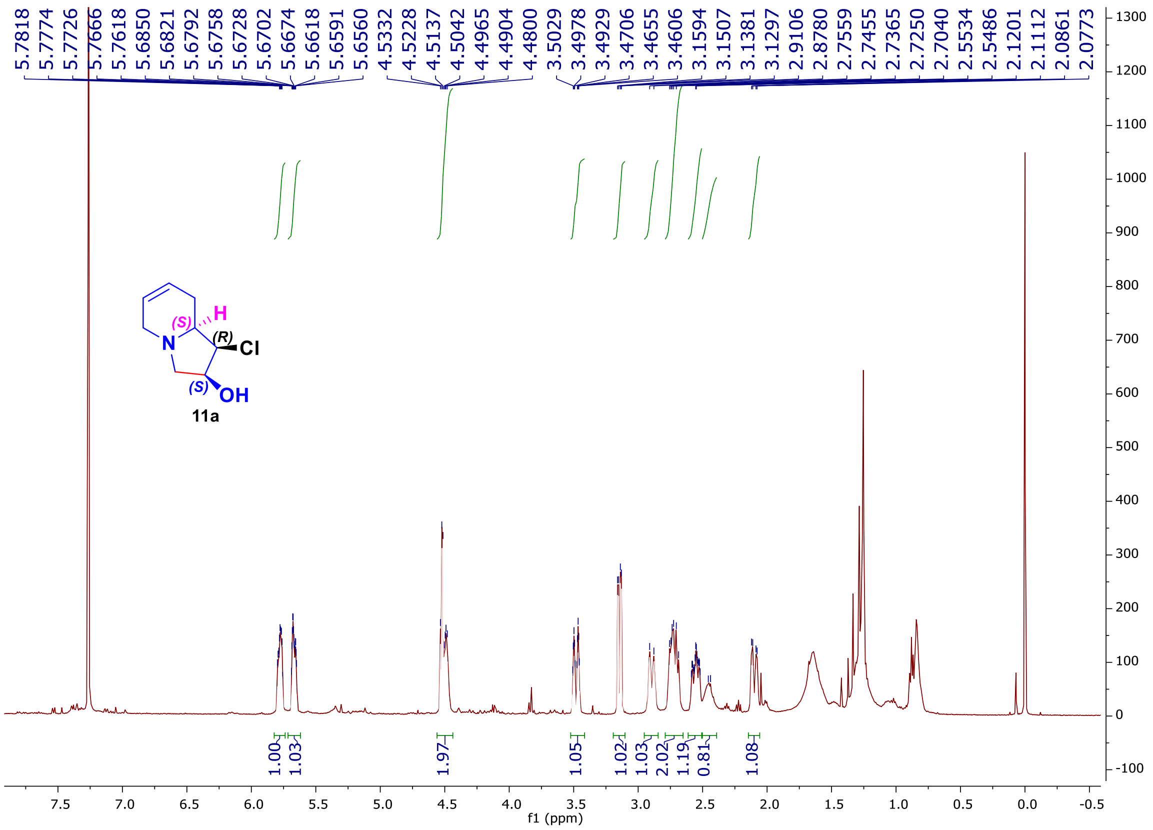
5.7818 5.7774 5.7726 5.7666 5.7618 5.6850 5.6821 5.6792 5.6758 5.6728 5.6702 5.6674 5.6618 5.6591 5.6560 4.5332 4.5228 4.5137 4.5042 4.4965 4.4904 4.4800 3.5029 3.4978 3.4929 3.4706 3.4655 3.4606 3.1594 3.1507 3.1381 3.1297 2.9106 2.8780 2.7559 2.7455 2.7365 2.7250 2.7040 2.5534 2.5486 2.1201 2.1112 2.0861 2.0773
(S)
(R)
Cl
(S)
OH
N
H
11a
1.00
1.03
1.97
1.05
1.02
1.03
2.02
1.19
0.81
1.08
f1 (ppm)

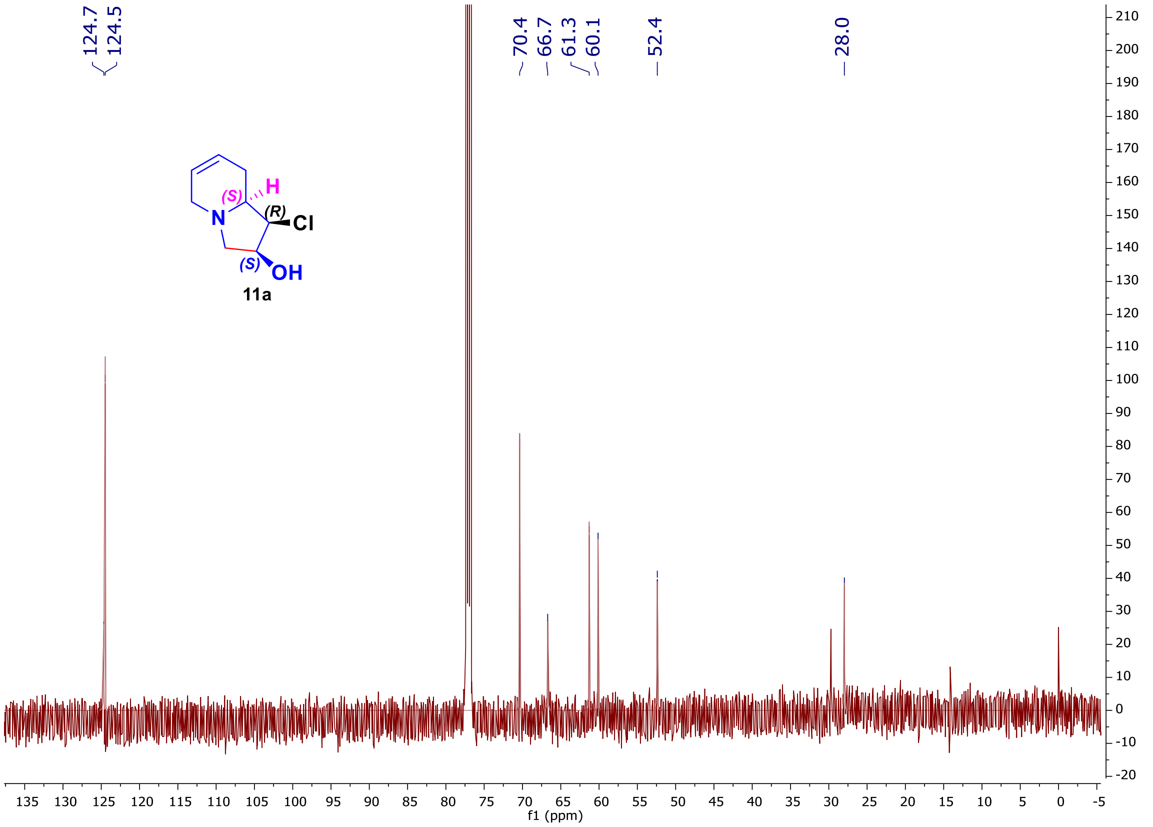
124.7
124.5
70.4
66.7
61.3
60.1
52.4
28.0
(S)
(R)
Cl
(S)
OH
N
H
11a
f1 (ppm)

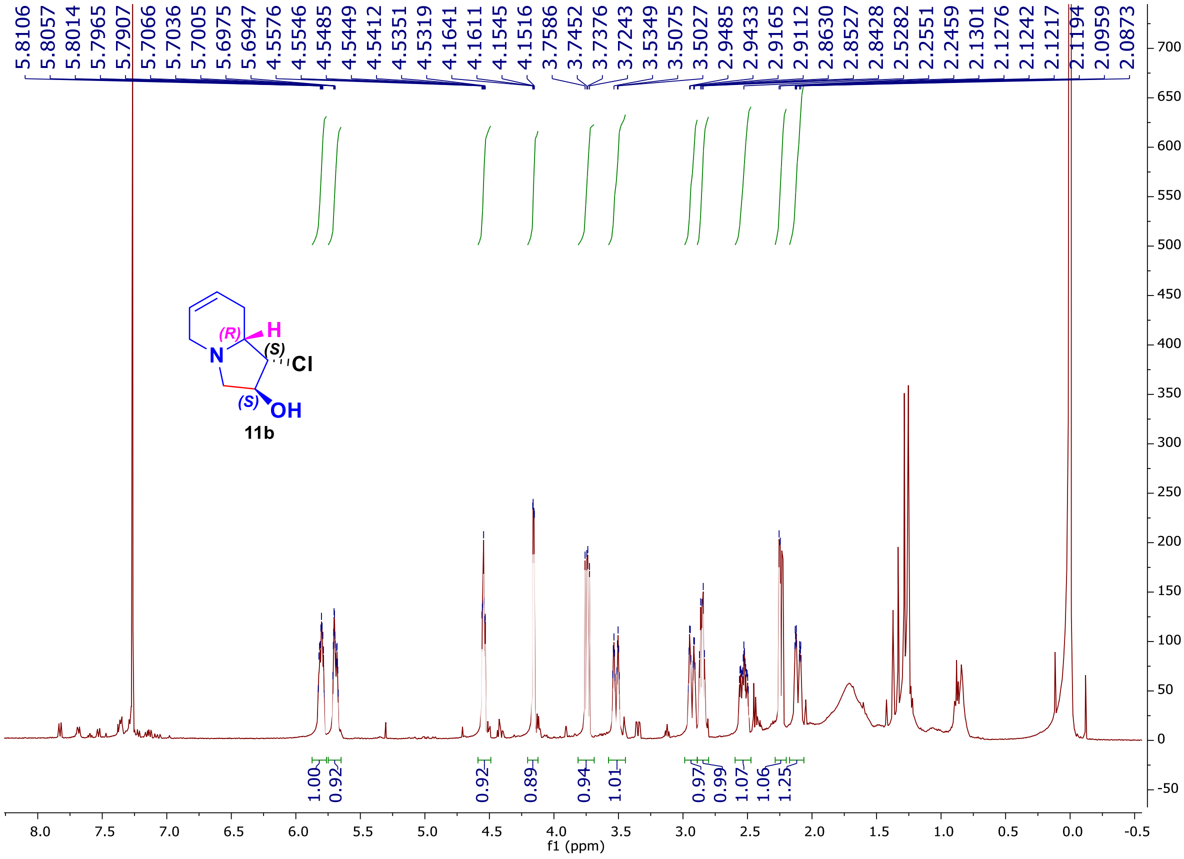
5.8106
5.8057
5.8014
5.7965
5.7907
5.7066
5.7036
5.7005
5.6975
5.6947
4.5576
4.5546
4.5485
4.5449
4.5412
4.5351
4.5319
4.1641
4.1611
4.1545
4.1516
3.7586
3.7452
3.7376
3.7243
3.5349
3.5075
3.5027
2.9485
2.9433
2.9165
2.9112
2.8630
2.8527
2.8428
2.5282
2.2551
2.2459
2.1301
2.1276
2.1242
2.1217
2.1194
2.0959
2.0873
(R)
H
(S)
Cl
N
(S)
OH
11b
1.00
0.92
0.92
0.89
0.94
1.01
0.97
0.99
1.07
1.06
1.25
f1 (ppm)

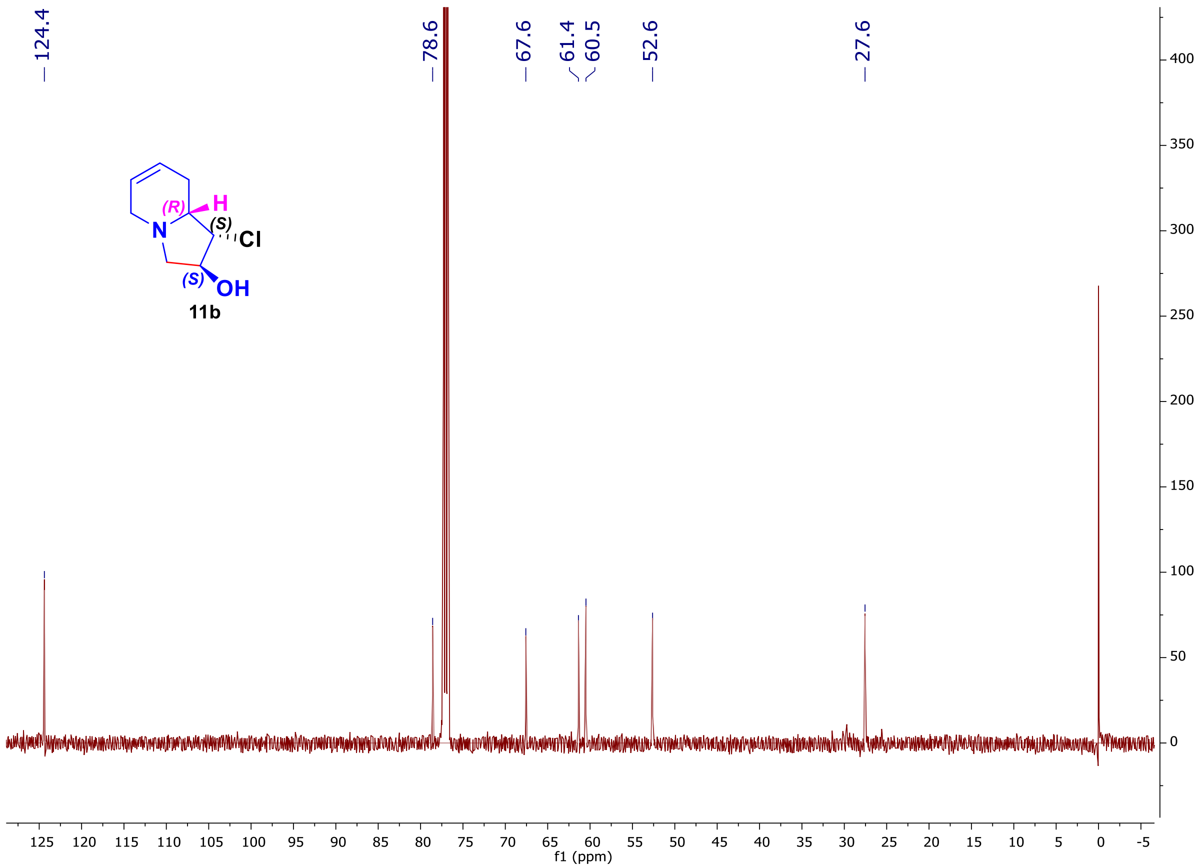
124.4
78.6
67.6
61.4
60.5
52.6
27.6
(R)
H
(S)
Cl
N
(S)
OH
11b
f1 (ppm)

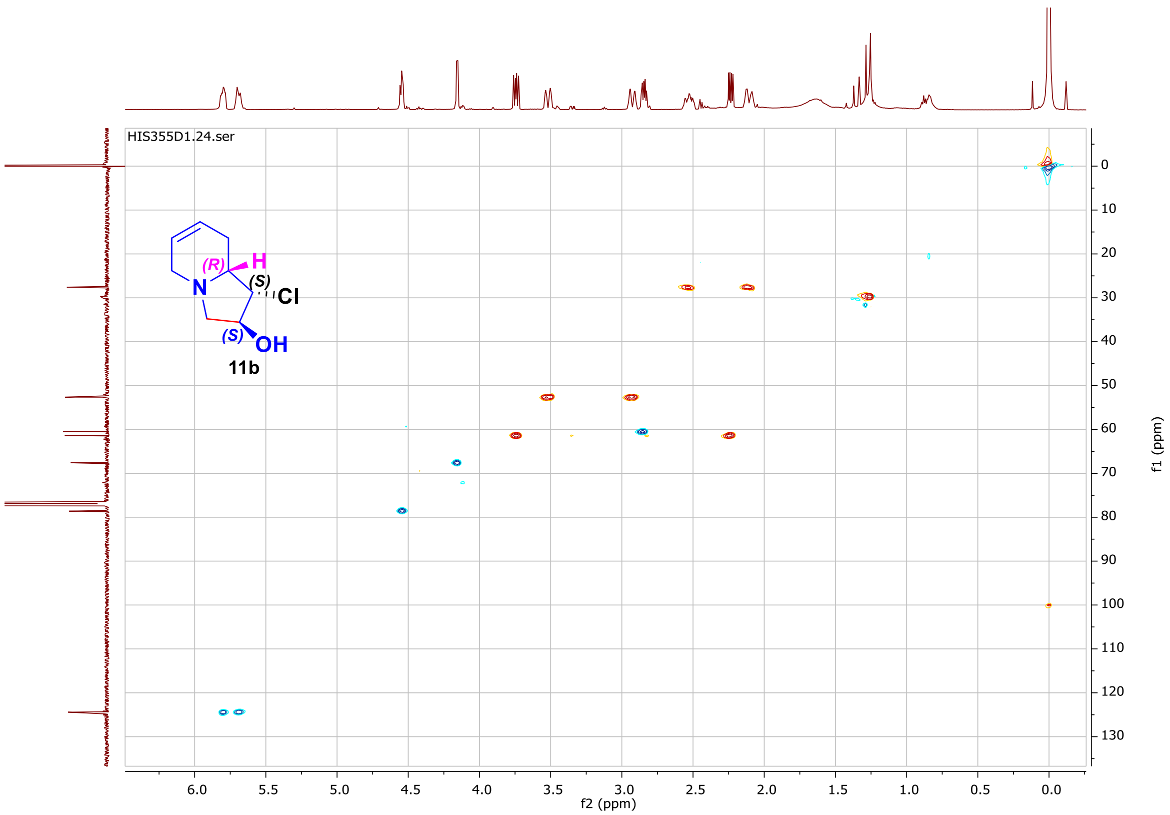
HIS355D1.24.ser
(R)
H
(S)
Cl
N
(S)
OH
11b
f2 (ppm)
f1 (ppm)

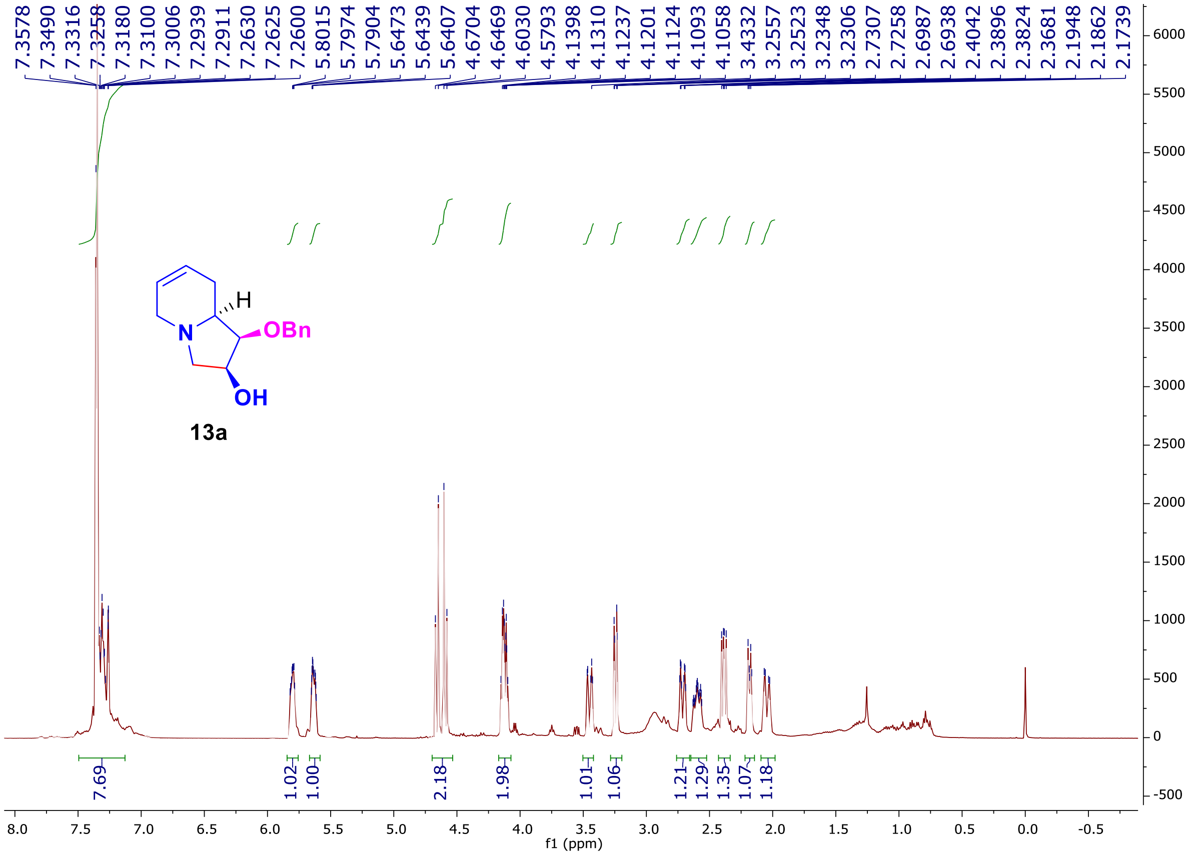
7.3578
7.3490
7.3316
7.3258
7.3180
7.3100
7.3006
7.2939
7.2911
7.2630
7.2625
7.2600
5.8015
5.7974
5.7904
5.6473
5.6439
5.6407
4.6704
4.6469
4.6030
4.5793
4.1398
4.1310
4.1237
4.1201
4.1124
4.1093
4.1058
3.4332
3.2557
3.2523
3.2348
3.2306
2.7307
2.7258
2.6987
2.6938
2.4042
2.3896
2.3824
2.3681
2.1948
2.1862
2.1739
H
N
OBn
OH
13a
7.69
1.02
1.00
2.18
1.98
1.01
1.06
1.21
1.29
1.35
1.07
1.18
f1 (ppm)

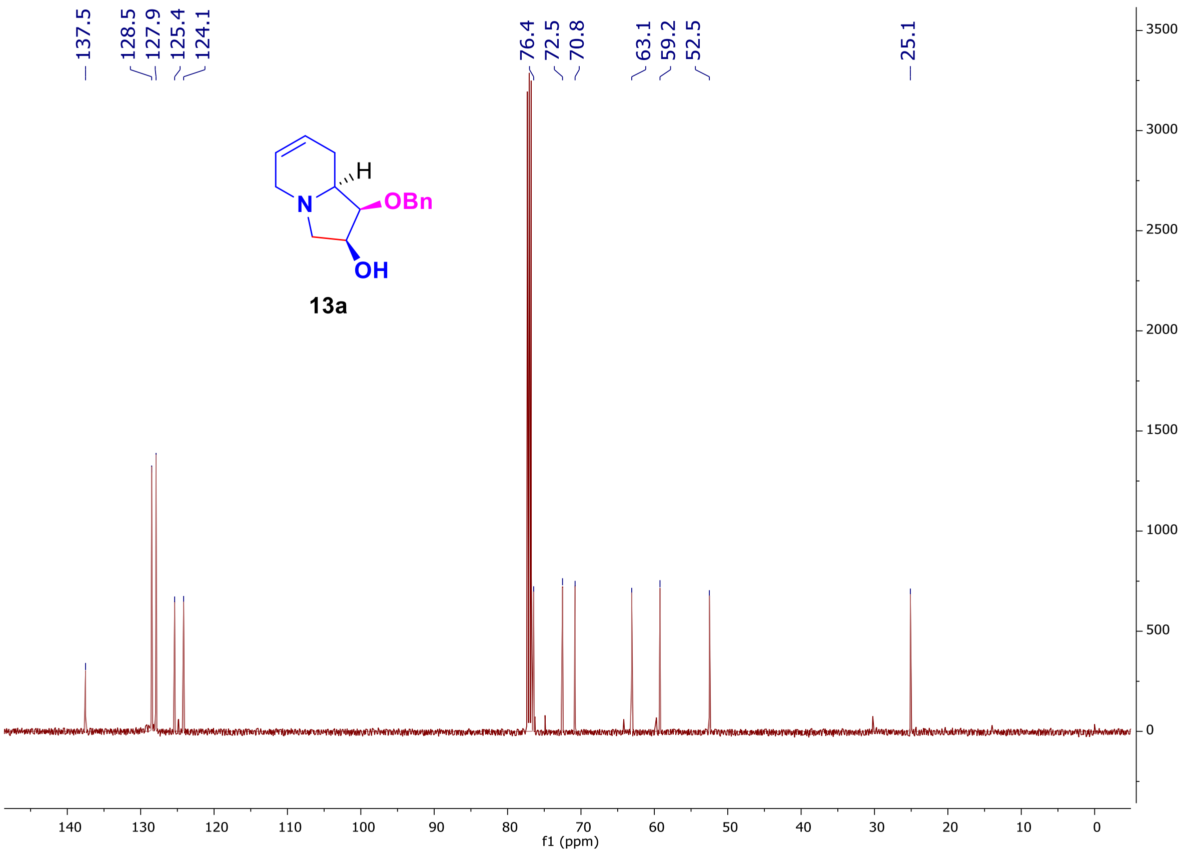

137.5
128.5
127.9
125.4
124.1
76.4
72.5
70.8
63.1
59.2
52.5
25.1
H
N
OBn
OH
13a
3500
3000
2500
2000
1500
1000
500
0
140 130 120 110 100 90 80 70 60 50 40 30 20 10 0
f1 (ppm)

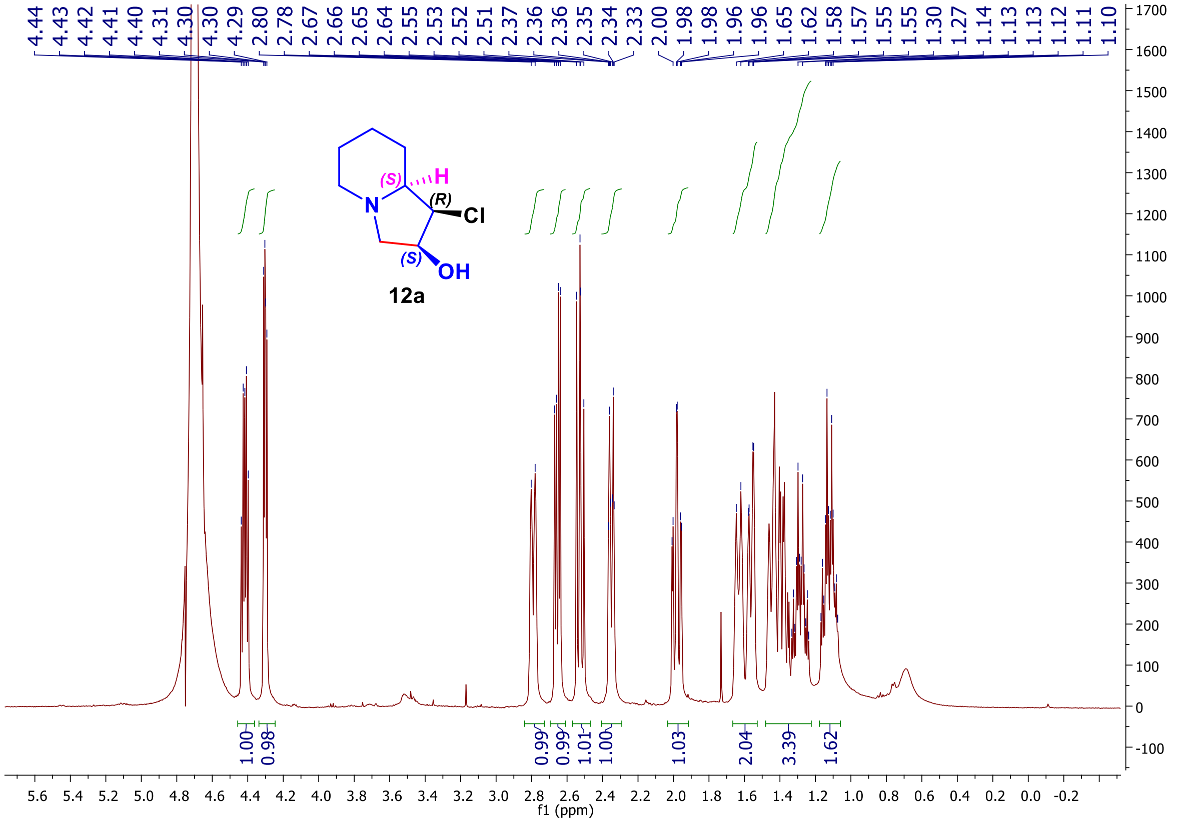
4.44 4.43 4.42 4.41 4.40 4.31 4.30 4.30 4.29 2.80 2.78 2.67 2.66 2.65 2.64 2.55 2.53 2.52 2.51 2.37 2.36 2.36 2.35 2.34 2.33 2.00 1.98 1.98 1.96 1.96 1.65 1.62 1.58 1.57 1.55 1.55 1.30 1.27 1.14 1.13 1.13 1.12 1.11 1.10
(S)
H
(R)
Cl
N
(S)
OH
12a
1.00 0.98 0.99 0.99 1.01 1.00 1.03 2.04 3.39 1.62
f1 (ppm)

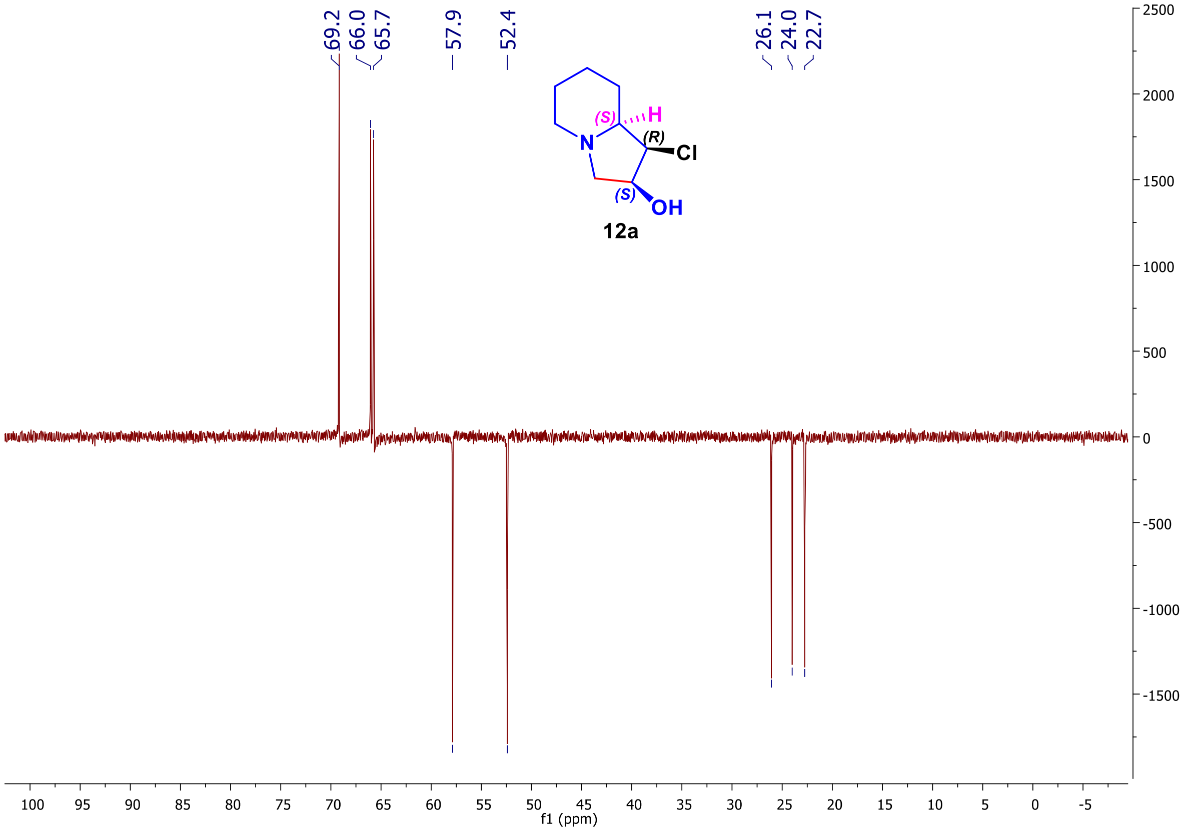
69.2 66.0 65.7 57.9 52.4 26.1 24.0 22.7
(S)
H
(R)
Cl
N
(S)
OH
12a
f1 (ppm)

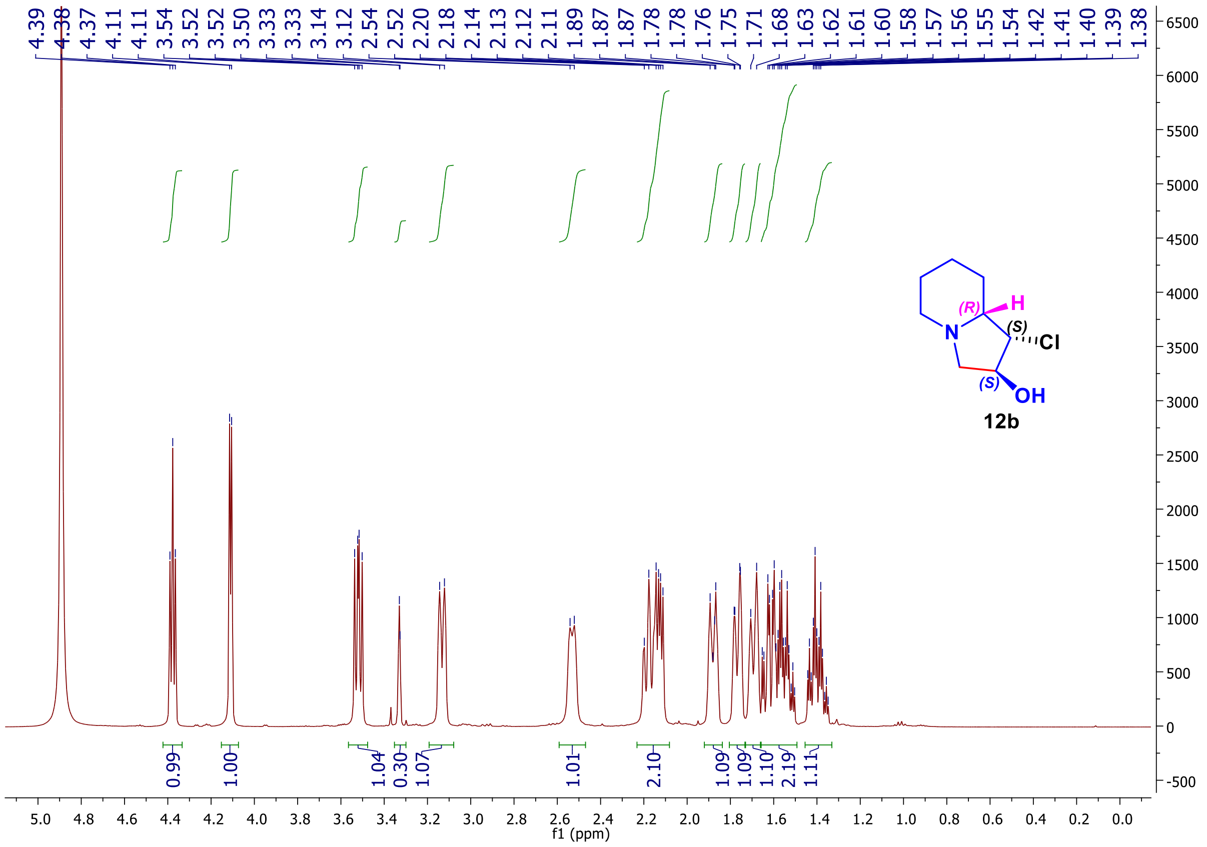
4.39 4.38 4.37 4.11 4.11 3.54 3.52 3.52 3.50 3.33 3.33 3.14 3.12 2.54 2.52 2.20 2.18 2.14 2.13 2.12 2.11 1.89 1.87 1.87 1.78 1.78 1.76 1.75 1.71 1.68 1.63 1.62 1.61 1.60 1.58 1.57 1.56 1.55 1.54 1.42 1.41 1.40 1.39 1.38
0.99 1.00 1.04 0.30 1.07 1.01 2.10 1.09 1.09 1.10 2.19 1.11
(R)
H
(S)
Cl
N
(S)
OH
12b
f1 (ppm)

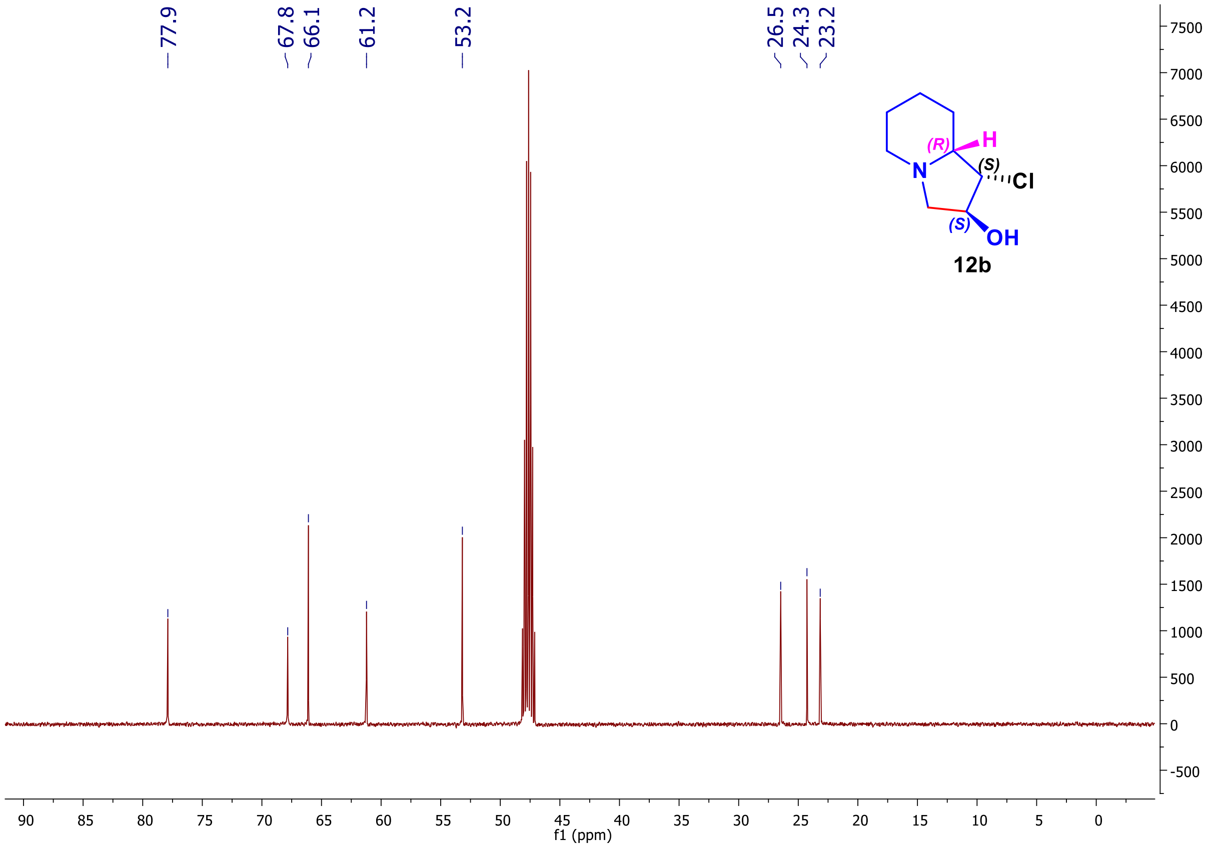
77.9 67.8 66.1 61.2 53.2 26.5 24.3 23.2
(R)
H
(S)
Cl
N
(S)
OH
12b
f1 (ppm)

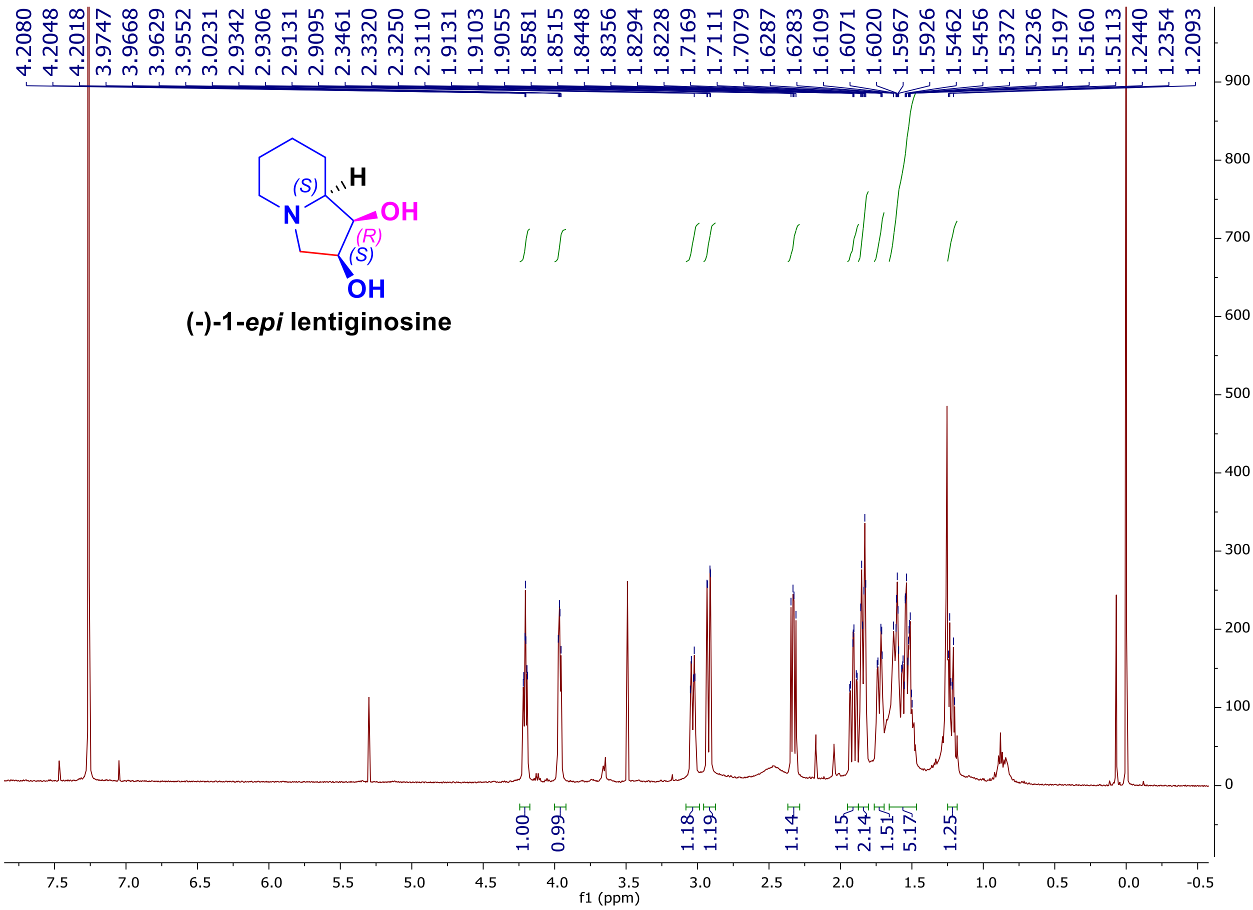
(-)-1-*epi* lentiginosine
f1 (ppm)

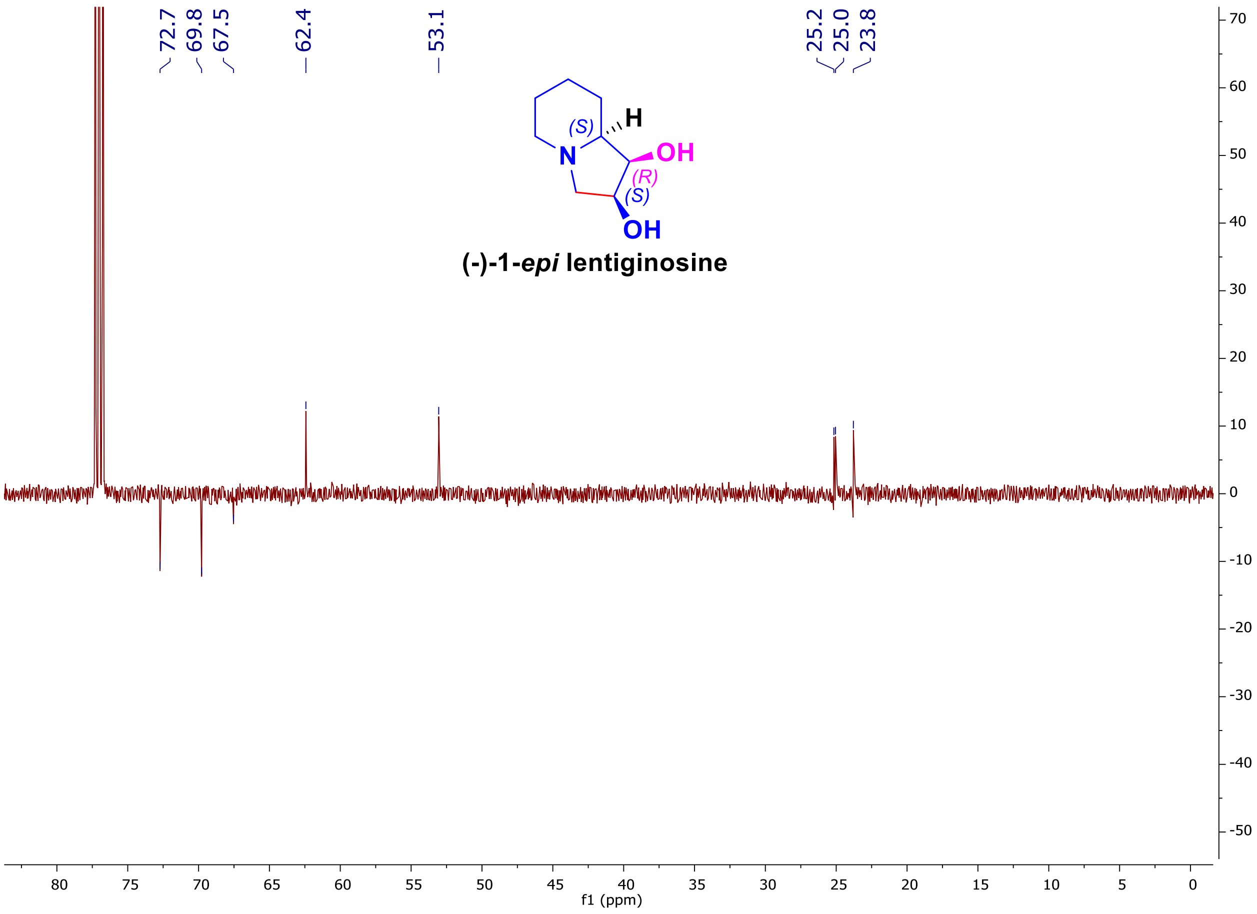
72.7
69.8
67.5
62.4
53.1
25.2
25.0
23.8
(S)
H
N
OH
(R)
(S)
OH
(-)-1-*epi* lentiginosine
f1 (ppm)

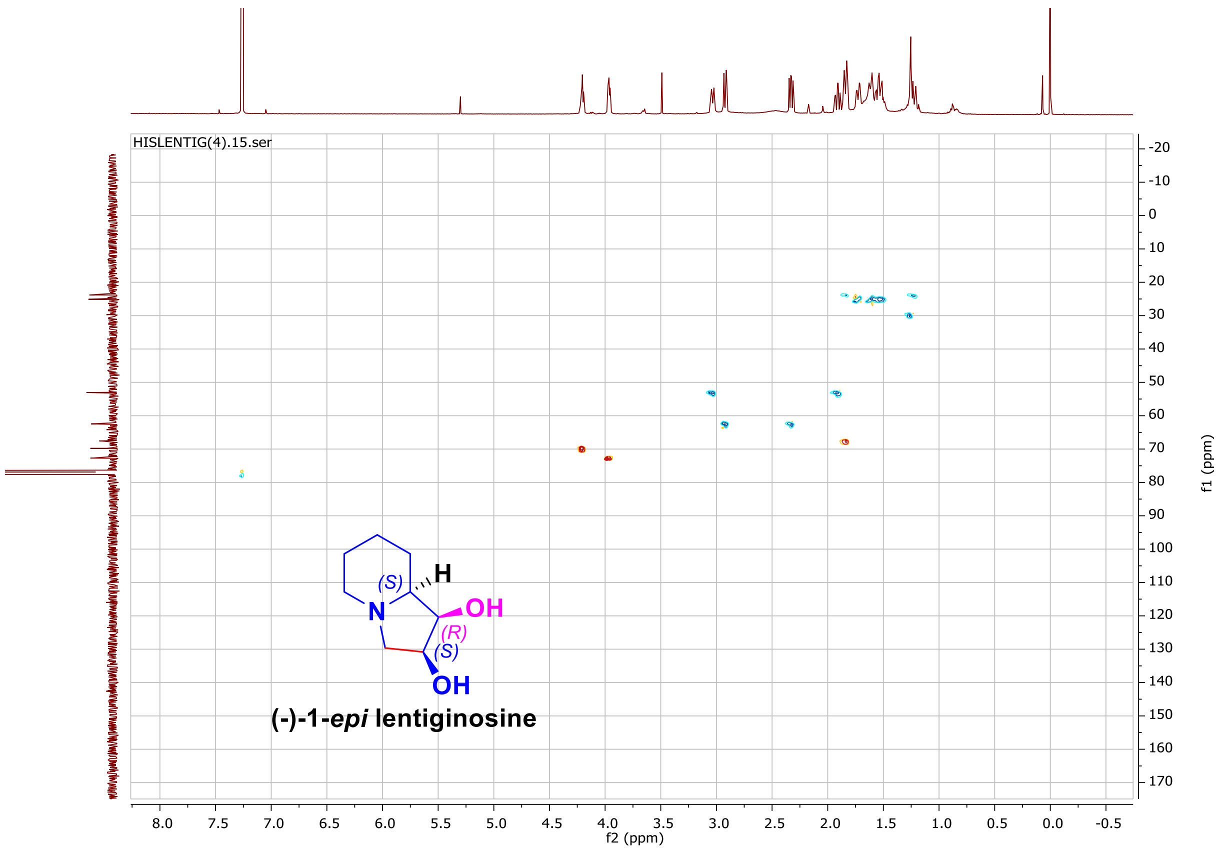
HISLENTIG(4).15.ser
f2 (ppm)
f1 (ppm)
(S)
H
N
OH
(R)
(S)
OH
(-)-1-*epi* lentiginosine

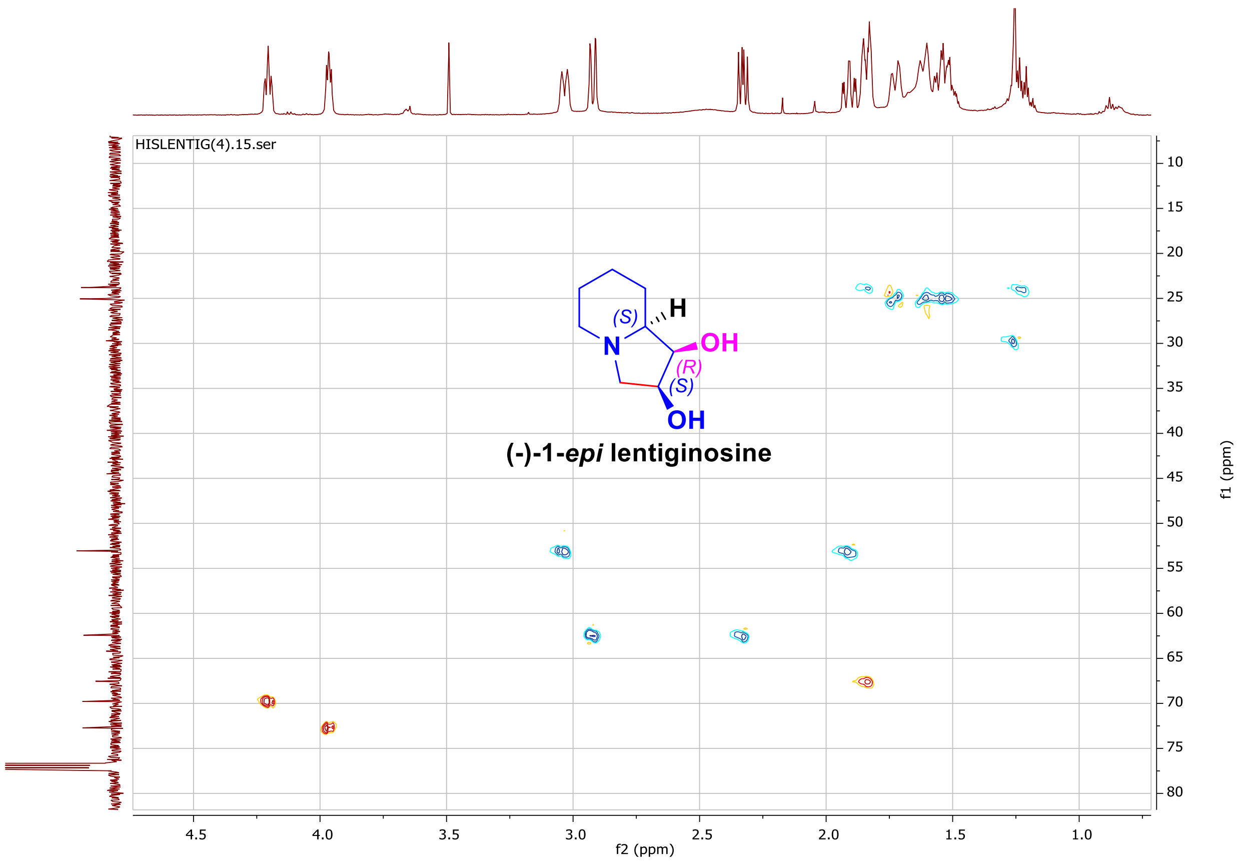
HISLENTIG(4).15.ser
(S)
H
N
OH
(R)
(S)
OH
(-)-1-*epi* lentiginosine
f2 (ppm)
f1 (ppm)

Table S1. Crystal data for compounds **3**, **11a**, **11b** and **12a**

	**3**	**11a**	**11b**	**12a**
CCDC deposition	2181510	2181511	2181512	2181513
Formula	$C_{20}H_{23}BrN_2O_3$	$C_8H_{12}ClNO$	$C_8H_{12}ClNO$	$C_8H_{14}ClNO$
fw	419.31	173.64	173.64	175.65
Crystal size (mm^3)	0.34×0.22×0.07	0.50×0.11×0.08	0.30×0.13×0.04	0.61×0.42×0.35
Colour	colourless	colourless	colourless	
Space group	$P2_12_12_1$	$P2_12_12_1$	$P2_12_12_1$	$P2_12_12_1$
a (Å)	7.1360(3)	7.3321(12)	10.8339(10)	7.4256(2)
b (Å)	8.9300(6)	10.641(2)	6.7230(9)	10.5444(4)
c (Å)	30.3879(14)	11.146(2)	11.3873(10)	11.2574(5)
V ($Å^3$)	1936.45(18)	869.6(3)	829.41(15)	881.44(6)
Z, *Z'*	4, 1	4, 1	4, 1	4, 1
Diffractometer	Stoe Stadivari	Stoe Stadivari	Stoe Stadivari	Stoe Stadivari
Radiation	Ag Kα	Ag Kα	Ag Kα	Ag Kα
T (K)	294(1)	294(1)	294(1)	295(1)
Density ($g.cm^{-3}$)	1.438	1.326	1.391	1.324
Absorption coef. (mm^{-1})	1.155	0.201	0.211	0.198
Transmission factors	0.5253 – 1.0000	0.5177 – 1.0000	0.4573 – 1.0000	0.6181 – 1.0000
Refl. collected (R_{int})	27808 (0.0741)	16944 (0.0715)	20777 (0.0761)	39302 (0.0230)
Refl. independent	4645	2040	1935	2656
(Senθ)/λ ($Å^{-1}$)	0.67	0.65	0.65	0.71
Data / parameters / restraints	4645 / 249 / 0	2040 / 103 / 0	1935 / 103 / 0	2656 / 105 / 0
Flack parameter	*x* = 0.034(13)	*x* = -0.01(7)	*x* = 0.01(6)	*x* = 0.028(15)
R_1, wR_2 [$I > 2\sigma(I)$]	0.0344, 0.0576	0.0311, 0.0528	0.0308, 0.0640	0.0295, 0.0794
R_1, wR_2 [all data]	0.1024, 0.0683	0.0638, 0.0590	0.0479, 0.0674	0.0335, 0.0841
GOF on F^2	0.817	0.784	0.864	1.066

X-ray intensities were collected at room temperature on a Stoe-Stadivari diffractometer equipped with an Axo microfocus source (Ag Kα radiation, λ = 0.56083 Å) and a Dectris Pilatus 100K detector [1]. Structures were refined with SHELXL [2]. For compound **3**, the O atom of the oxazolidine ring is disordered over two positions, O3A and O3B, placed above and below the mean plane of this heterocycle. Occupancies for these sites were refined to 0.37(2) and 0.63(2) for O3A and O3B, respectively. No disordered parts were detected in the other compounds. For all compounds, the refined Flack parameter is consistent with the absolute configuration expected from the synthetic procedure. In all cases, all H atoms bonded to C atoms were placed in calculated positions, while hydroxy H atoms positions were determined from difference maps, and refined with free coordinates. All isotropic displacement parameters for H atoms are calculated from the equivalent displacement parameters of their carrier C/O atoms. Structure factors can be found in the Cif files deposited with the CCDC. Checkcif/PLATON reports are copied below, which do not raise A/B-level issues.

[1] Stoe & Cie (2019). X-AREA and X-RED32. Stoe & Cie, Darmstadt, Germany.
[2] Sheldrick, G. M. (2015). *Acta Cryst*. C**71**, 3-8.

Datablock 3_Bromohydrin - ellipsoid plot

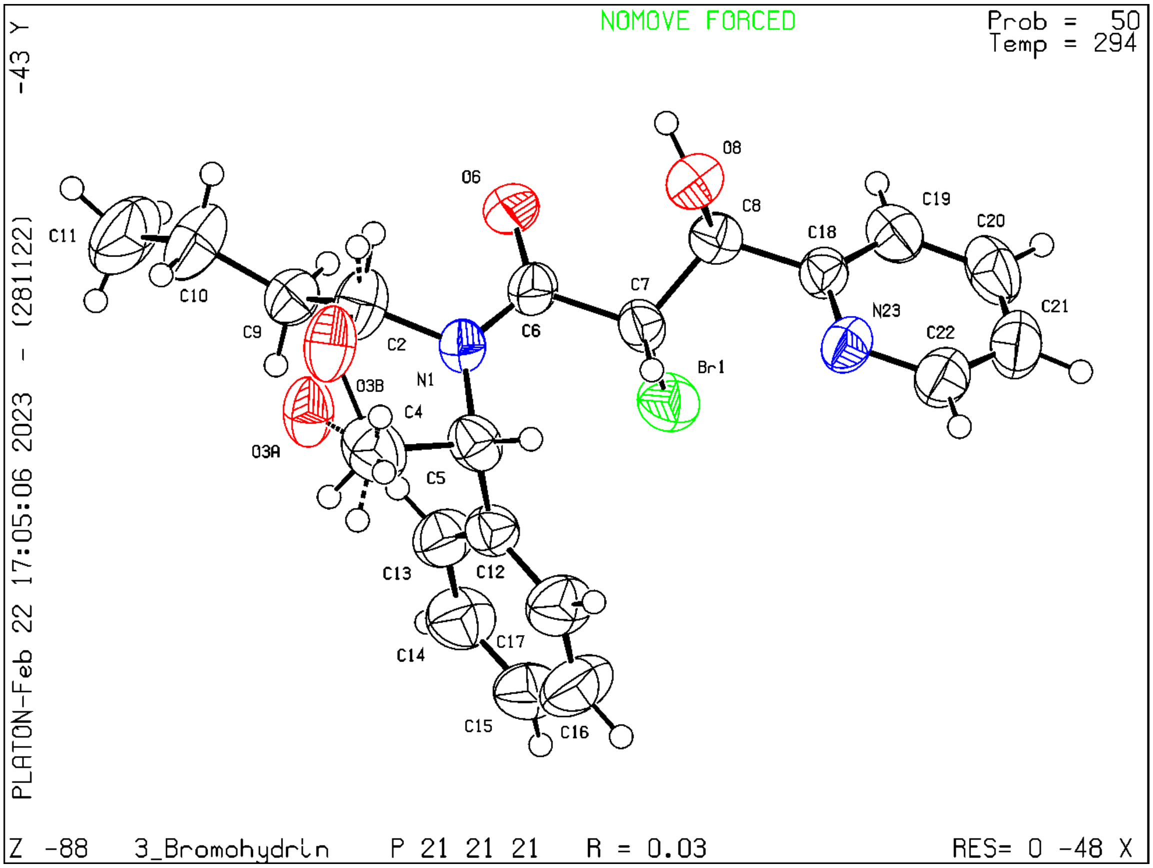

Datablock 11a_Delta4-cis - ellipsoid plot

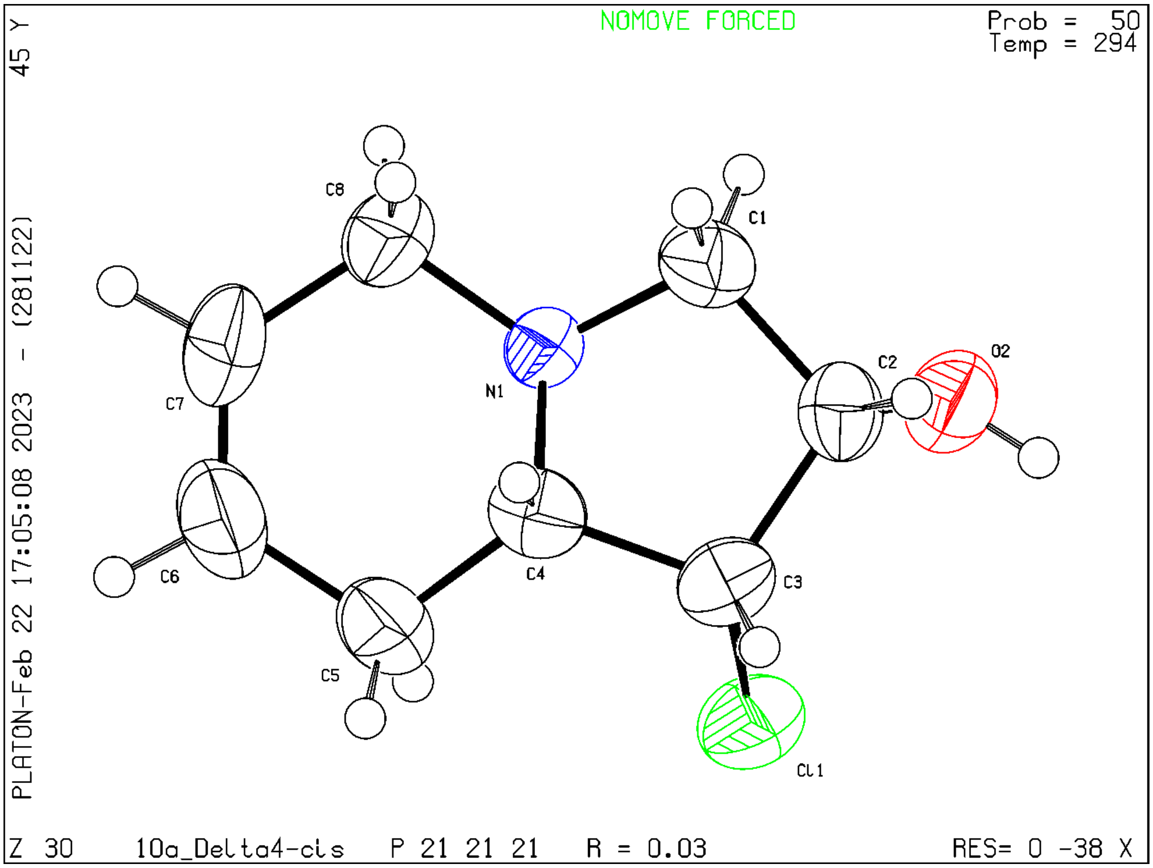

Datablock 11b_Delta4-trans - ellipsoid plot

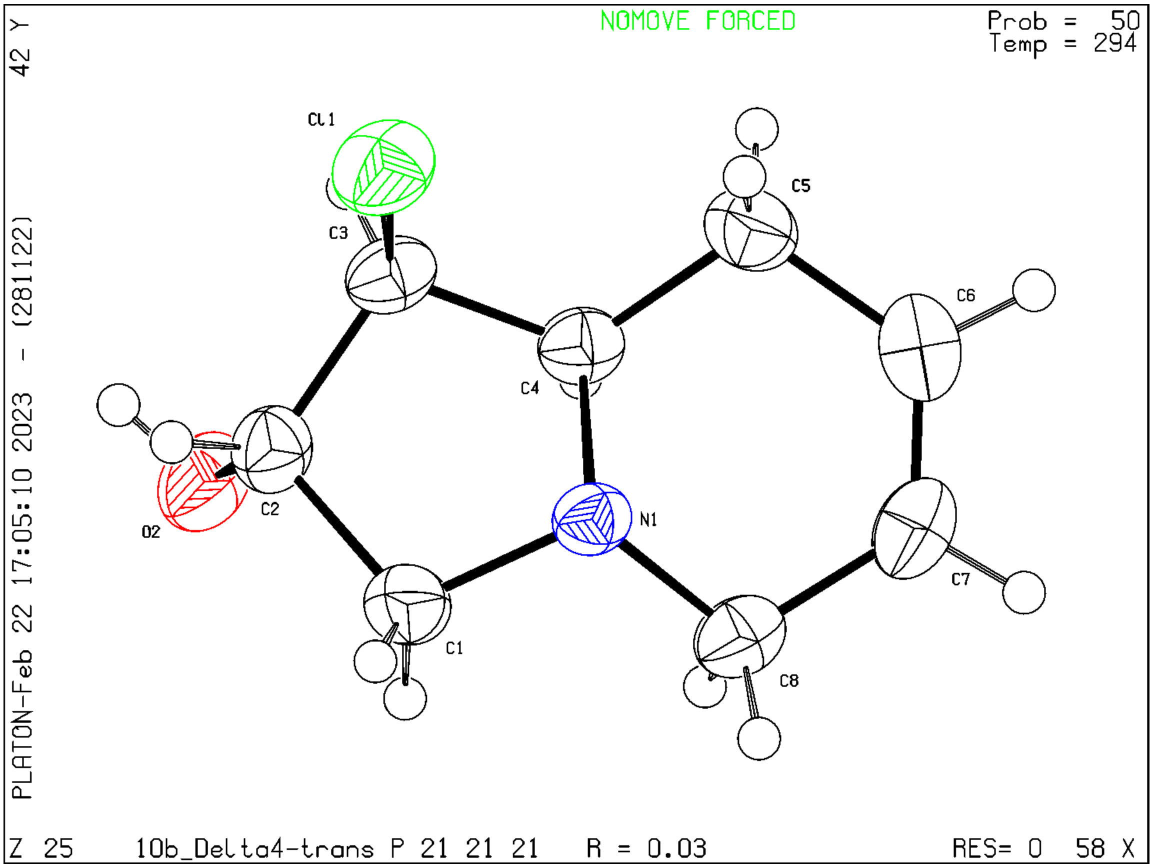

Datablock 12a_Indolizine-cis - ellipsoid plot

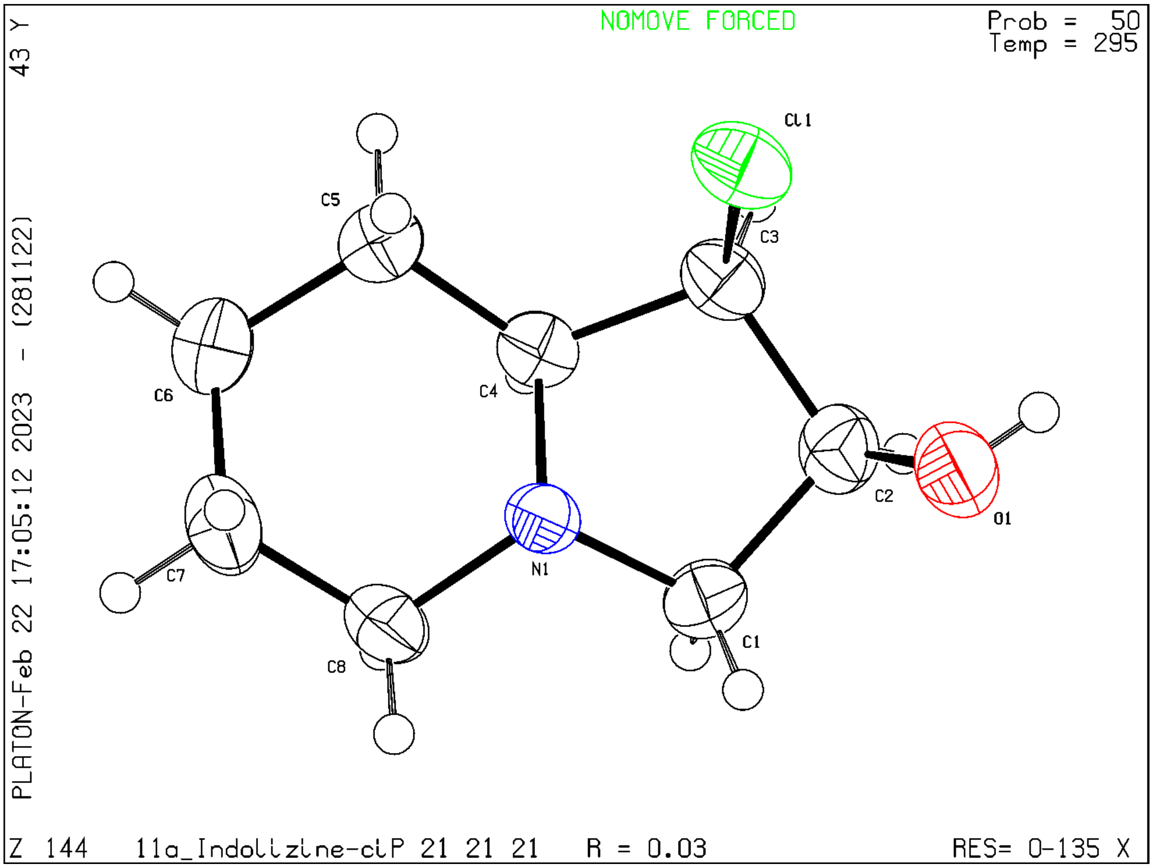

Supplement: Supplementary file 1 [file molecules-28-03719-s001.zip › molecules-2362226-supplementary.pdf]
